# Redox chemistry, catalysis, and electrochemical detection of periodate species: sensors, mechanisms, and applications in green chemistry

**DOI:** 10.3389/fchem.2026.1881498

**Published:** 2026-08-25

**Authors:** Ruba Alrajhi, Fatehy M. Abdel-Haleem, Mikhael Bechelany, Ahmed Barhoum

**Affiliations:** 1 Department of Chemistry, College of Science, Imam Mohammad Ibn Saud Islamic University (IMSIU), Riyadh, Saudi Arabia; 2 Institut Européen des Membranes, IEM, UMR 5635, University Montpellier, ENSCM, CNRS, Montpellier, France; 3 Department of Mathematics and Natural Sciences, Gulf University for Science and Technology (GUST), Hawally, Kuwait; 4 School of Chemical and Biopharmaceutical Sciences, Technological University Dublin, Dublin, Ireland; 5 Nanolab Research Centre, Physical to Life Sciences Research Hub, Technological University Dublin, Dublin, Ireland

**Keywords:** amperometry, catalytic oxidation, electrochemical sensing, green chemistry, nanomaterials, periodate, potentiometry, voltammetry

## Abstract

Periodate species, as potent oxidizing agents, are widely applied in environmental remediation, pharmaceutical synthesis, analytical chemistry, and advanced oxidation processes due to their superior redox and catalytic behaviors. Nowadays, periodate-mediated reactions are drawing more attention as a green and sustainable synthetic methodology in active pharmaceutical ingredients (APIs) synthesis, organic pollution degradation, and fine chemicals transformation. As a result, developing sensitive, selective, and rapid analytical methods for periodate determination has gained significant attention. This review summarizes a detailed analysis of the chemistry, redox behavior, catalysis properties, and electrochemical determination of periodate species. A special emphasis is given to the most recent research progress on electrochemical sensing platforms for direct and indirect determination of periodate and periodate-reactive compounds, including alcohols, carbohydrates, amines, and amino acids, among others. In addition, electrochemical methods used for the determination of transition-metal catalysts in periodate-mediated catalytic systems are also included. Different electrochemical sensing platforms such as ion-selective membrane electrode, nanomaterial-modified glassy carbon electrode, noble-metal electrode, and perchlorate selective electrode are discussed, respectively, in combination with potentiometric, amperometric, and voltammetric techniques and analyzed in aspects of sensitivity, selectivity, detection limits, stability, and practicability. Additionally, the significance of periodate in green chemistry and wastewater treatment is described. Moreover, the challenges, mechanistic interpretations, and future outlook toward high-performance, portable, and nanostructured electrochemical sensors are presented.

## Graphical Abstract

**Figure:**
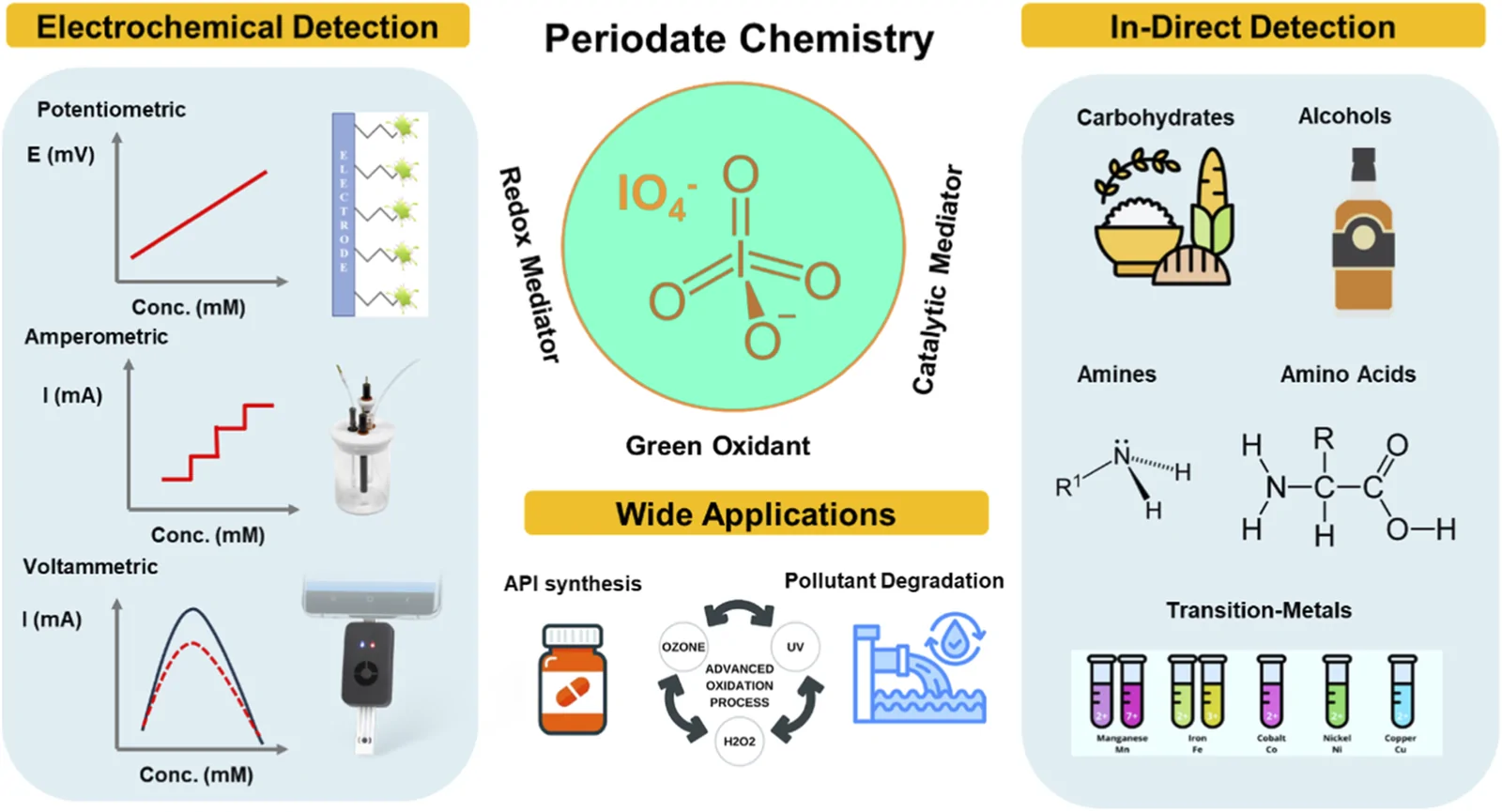

## 1 Introduction

Periodate anion (IO; PIT) is an environment-friendly and chemically and biologically significant oxidizing species that exhibits robust oxidation and catalytic capacity. PIT is a member of the high-valence iodine oxyanions family and is comprised of a single iodine atom bonded to four surrounding oxygen atoms in a tetrahedral arrangement. With its high oxidation potential and selectively active nature, PIT takes part in a wide variety of organic and inorganic reactions such as oxidative cleavage, iodination, oxidation of alcohols, and decomposition of pollutants (Equation 1) (Arndt et al., 2022a; Chen et al., 2022; Kisukuri et al., 2022).

$${\text{IO}}_{4}^{-}+B\;\to \;{\text{IO}}_{3}^{-}+\text{product}.$$ (1)

PIT has been the focus of significant interest and study due to its unique property of efficiently oxidizing diverse substrates under reasonably mild conditions, which is important for analytical chemistry, pharmaceutical synthesis, environmental remediation, and green chemistry. PIT-mediated reactions have been widely used for the synthesis of fine chemicals and active pharmaceutical ingredients (APIs), treatment of pharmaceutical wastes, and oxidation of pollutants in advanced oxidation processes (AOPs) (Arndt et al., 2022a; Chen et al., 2022; Leguy et al., 2018; Mecozzi et al., 2019). PIT offers significant advantages such as the nature of oxidation being environmentally benign compared to many conventional oxidants and having lower toxicity, no hazardous byproducts, and high storage stability, among others. However, limitations on wider applications of high-grade PIT are mainly due to its expensive synthesis, scarce commercial supply with high purity, and the lack of quick and sensitive analytical techniques for its analysis and control (Arndt et al., 2020).

Significant advances have been achieved in the electrochemical synthesis, analytical determination, and electrocatalytic applications of PIT. Electrochemical methodologies provide a fast, controllable, and environmentally benign route for periodate generation, while also enabling its integration into catalytic oxidation systems. Owing to its strong oxidizing capability, periodate has been widely utilized in both chemical and electrochemical transformations, such as the conversion of hydroquinones to quinones, hydrazoarenes to azoarenes, and vicinal diols to dialdehydes (Arndt et al., 2022a; Sudalai et al., 2015). The advantages of using electrochemistry in the application for PIT are good sensitivity, simplicity, rapidity, portability, and low consumption of reagents, and it can also be used for online determination. Different electrochemical detection methods such as potentiometry, amperometry, and voltammetry were used for direct determination of PIT and for the indirect analysis of other species through a PIT-mediated redox reaction. Several sensing platforms such as PIT selective membrane electrodes, nanomaterial-modified glassy carbon electrodes, noble-metal electrodes, carbon-based electrodes, and perchlorate selective electrodes have been developed. Various nanostructured materials such as nano-metallic materials, graphene derivatives, and conducting polymers were developed to boost the electrocatalytic activity and improve the electron-transfer kinetics, sensitivity, and selectivity toward PIT and the relevant species.

Although some literature had described the chemistry, oxidation of PIT, and its catalysis application (Arndt et al., 2022a; Efstathiou and Hadjiioannou, 1995; Mecozzi et al., 2019), the analysis and electroanalysis using PIT have not been reviewed in details. In this review, we highlight the synthesis, redox chemistry, and catalytic activities of PIT and the close relationship with the electrochemical determination and electroanalytical sensing of PIT. This review systematically compares the analytical performance and various electrochemical analytical techniques, sensing material, and electrode-modification strategies used for both direct and indirect determination of PIT. Furthermore, recent advancements using PIT in the pharmaceutical industry, industrial application, environment protection, and green chemistry field have been summarized together with the research challenges, current development and opportunities of sensor design, and green analytical science, covering 105 references with recent references.

## 2 Redox chemistry and catalysis of periodate

### 2.1 Structural diversity and physicochemical properties of periodate species

Iodine can form different oxyanions with different oxidation states, structures, and oxidation properties. These oxyanions include, amongst others, the iodide species with an oxidation state of one and the periodate species with iodine at its maximum oxidation state (+7), which is listed in Table 1. The outstanding redox and catalytic properties of PITs compared to that of other iodine-containing oxyanions are largely attributed to the highly electron-deficient central iodine atom coupled with the possession of various oxygen atoms that are capable of initiating electron transfer reactions (Arndt et al., 2022a; Chen et al., 2022; Efstathiou and Hadjiioannou, 1995). PIT compounds occur in several structural forms ranging from discrete monomers (e.g., NaIO and KIO) to hydrated, condensed, and polymeric structures such as KIO, Pb(IO), CuIOHO, and NaHIO, among others (Table 1) (Efstathiou and Hadjiioannou, 1995). The diverse range of structures arises from the acidic nature of periodic acid and the extensive tendency of this species to hydrate, condense, and polymerize in an aqueous medium (Efstathiou and Hadjiioannou, 1995). The diverse nature of the different structures has profound impacts on the various physicochemical properties, including their solubility, thermal stability, ionic conductivity, redox potential, and catalytic activity and, hence, have a variety of applications in electrocatalysis, analytical chemistry, environmental treatment, and pharmaceutical preparation, among are (Efstathiou and Hadjiioannou, 1995; Lee and Yoon, 2004; Parent et al., 2012; Sahu and Mishra, 2020; Sklarz, 1967).

**TABLE 1:** Chemical formulas, nomenclature, oxidation states, and representative structures of iodine oxyanions and selected periodate species (Efstathiou and Hadjiioannou, 1995; Sklarz, 1967).

Formula	Nomenclature	Iodine oxidation state	Representative structures
KI	Iodide	−1	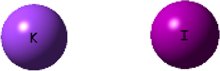
KIO	Hypoiodite	+1	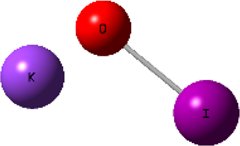
KIO_2_	Iodite	+3	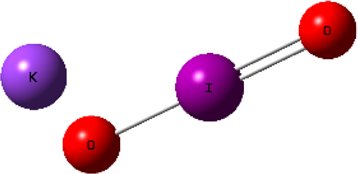
KIO_3_	Iodate	+5	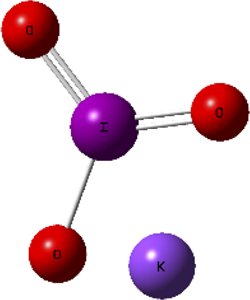
NaIO_4_	Sodium metaperiodate	+7	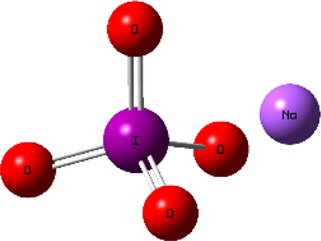
K_4_I_2_O_9_	Potassium dimesoperiodate	+7	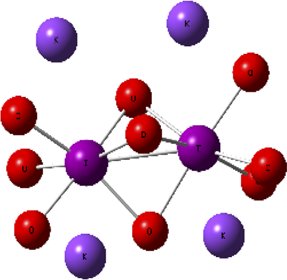
Pb_3_(IO_5_)_2_	Lead metaperiodate	+7	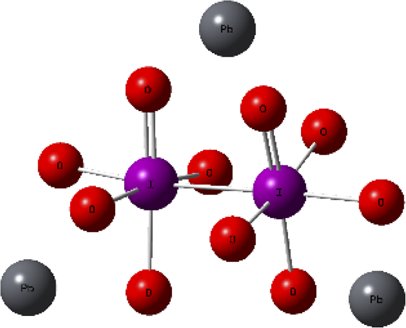
Cu_4_I_2_O_11_·H_2_O	Copper periodate monohydrate	+7	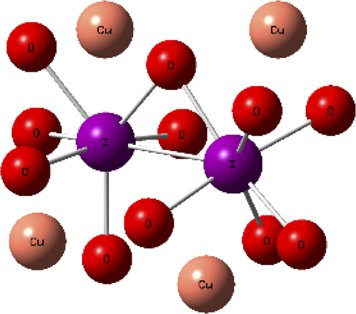
Na_3_H_2_IO_6_	Sodium paraperiodate	+7	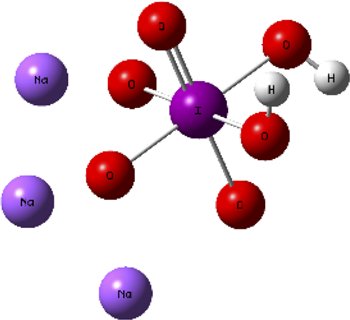
Y_4_I_2_O_13_·11H_2_O	Yttrium periodate undecahydrate	+7	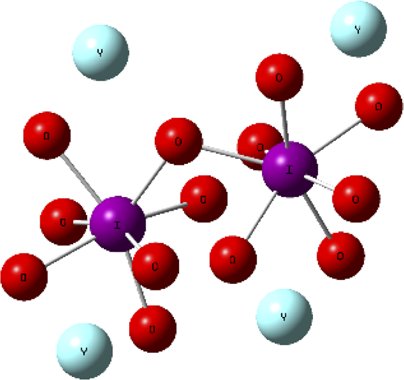

An outstanding chemical feature of PIT is the ability to selectively cleave the carbon–carbon bonds in vicinal diols and polyhydroxylated compounds under mild conditions. The process, known as Malaprade reaction (Equations 2, 3), is accompanied by the rapid formation of cyclic periodate esters followed by cleavage of the C–C bond, yielding aldehydes, ketones, or carboxylic derivatives depending on the structure of the substrates and reaction conditions (Arndt et al., 2022a; Efstathiou and Hadjiioannou, 1995; Mecozzi et al., 2019; Sklarz, 1967; Sudalai et al., 2015; Wong and Shing, 2014).

$$>C\left(OH\right)-C\left(OH\right)<+IO_{4}\to \;>C=O+H_{2}O+>C=O+IO_{3}^{-}.$$ (2)

$$>C\left(OH\right)-{\left(CHOH\right)}_{n}-C\left(OH\right)<+\left(n+1\right)IO_{4}\to$$

$$>C=O+nHCOOH+>C=O+\left(n+1\right)IO_{3}^{-}+H_{2}O.$$ (3)

**FIGURE 1:**
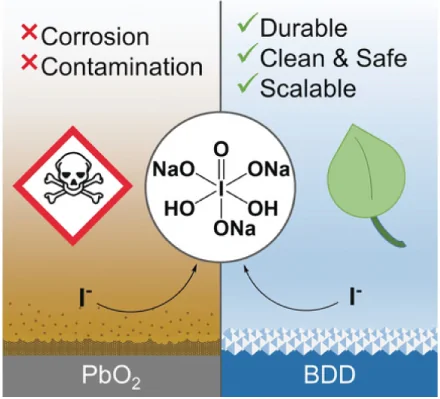
Schematic illustration of electrochemical periodate production from iodide, comparing the boron-doped diamond (BDD) and conventional PbO_2_ anodes for efficient periodate generation (Arndt et al., 2020).

This reaction is normally fast, stoichiometric, irreversible, highly selective, and, hence, very useful in carbohydrate chemistry, structural elucidation of polysaccharides, biochemical analysis, pharmaceutical synthesis, and analytical chemistry. Oxidation of alcohols, amino alcohols, glycols, and bio-active compounds can also be mediated using PIT oxidation, which enables site-selective functionalization. Most recently, the application of PIT in the degradation of pharmaceuticals pollutants and dyes and the activation of advanced oxidation processes in water treatment have been reported, and this indicates PIT is a green oxidant (Figure 1) (Lee and Yoon, 2004; Parent et al., 2012; Sklarz, 1967).

Even though PIT has been widely used in synthetic chemistry and oxidation processes, certain intrinsic chemical features remain poorly understood, such as the behavior of PIT in complex aqueous matrices, the electrocatalytic mechanism of the oxidation, the intermediate radical species, and the interaction with nanostructured electrode surface materials. Comparative studies on relationships between the specie formulation of PIT, its oxidation potential, catalytic activity, and sensing behavior are limited in the literature, with most literature being focused on the synthetic aspects and oxidation mechanism rather than the relationship with electrochemical performance. The comprehensive study of the forms and equilibria of PIT, its physiochemical properties, and its oxidation chemistry are a scientific foundation to discuss its electrochemistry, electrocatalysis, and analytical applications.

### 2.2 The effect of pH on the periodate species and oxidation activity

**FIGURE 2:**
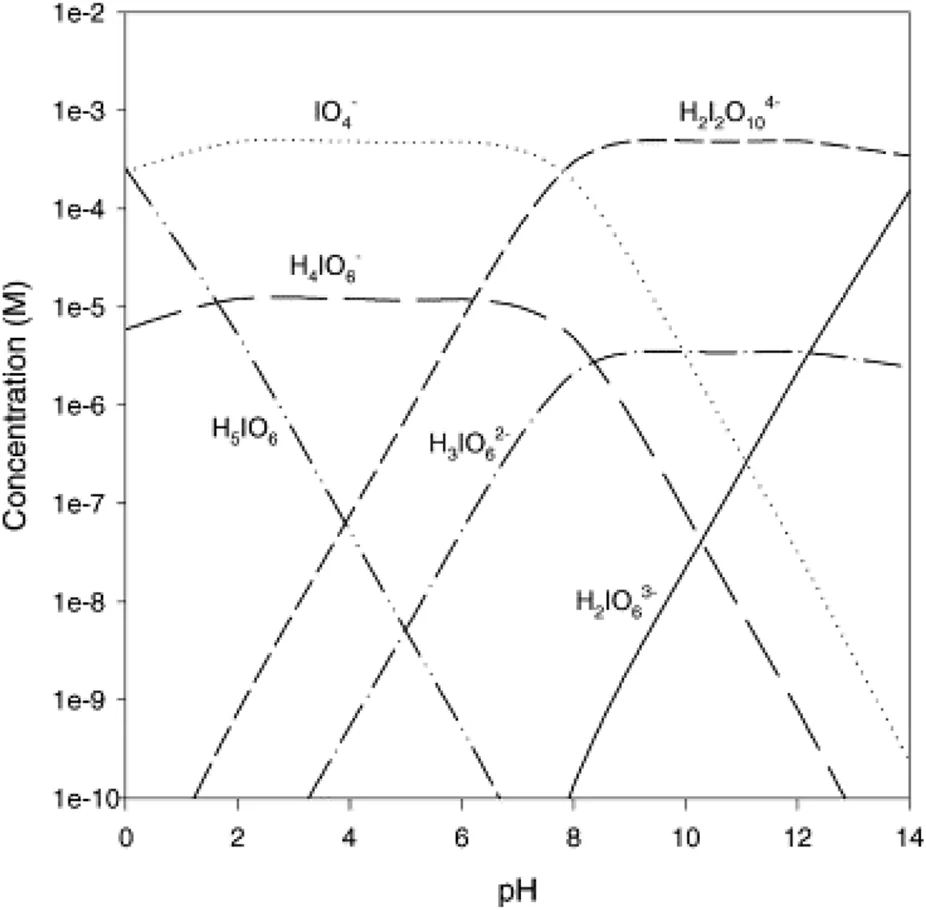
Effect of pH on the distribution of PIT species, showing the equilibrium among the protonated, deprotonated, and dimerized forms and its influence on their oxidation potential, stability, and electrochemical activity (Lee and Yoon, 2004).

The oxidation nature and chemical reactivity of periodate ions are greatly influenced by the pH of the medium since periodic acid undergoes several acid–base and hydrolysis equilibria in the aqueous solution. The solution chemistry of PIT is particularly complicated because the periodate species can exist in several different protonated, hydrated, and dimerized states depending on the pH, ionic strength, concentration, and temperature. Orthoperiodic acid, HIO, is the most thermodynamically stable form; it behaves as a tribasic acid such as orthophosphoric acid with stepwise proton removal, as shown in Equation 4 (Chen et al., 2022; Efstathiou and Hadjiioannou, 1995).

$$H_{5}{\text{IO}}_{6}\;⇌\;H_{4}{\text{IO}}_{6}^{-}+H^{+}\;K_{1}=5.1\times 10^{-4}$$

$$H_{4}{\text{IO}}_{6}^{-}\;⇌\;H_{3}{\text{IO}}_{6}^{2-}+H^{+}\;K_{2}=2.0\times 10^{-7}$$

$$H_{4}{\text{IO}}_{6}^{-}\;⇌\;{\text{IO}}_{4}^{-}+2H_{2}O\text{ KD}=40\;\left(25\;{}^{\circ}C\right),$$ (4)

where K_1_ = 5.1 × 10^−4^, K_2_ = 2.0 × 10^−7^ and K_D_ = 40 (at 25 °C). The stability of the IO_4_^−^ solutions was confirmed in the absence of light, and its diluted solutions (1 × 10^−2^ to 5 × 10^−2^ M) are kept in dark bottles and away from the organics or oxidizable species (Efstathiou and Hadjiioannou, 1995). The acid–base equilibria of the periodic acid play a vital role in determining the redox behavior, oxidation kinetics, and electrochemical response of the PIT species. While the monomeric species such as IO in the acidic and slightly acidic pH regions are strong oxidizing species, in the alkaline media, dimerization and hydration lead to the formation of orthoperiodate dimers such as HIO that have lower oxidation potential and oxidation capacity (Figure 2) (Efstathiou and Hadjiioannou, 1995; Lee and Yoon, 2004). This implies that controlling the pH of the solution is very critical for the application of PIT oxidation and its electrochemical sensing. PIT solutions are generally stable in diluted aqueous medium when protected from light and organic contamination. The photosensitivity of PIT may lead to decomposition of the solution, with the formation of ozone and reactive oxygen species (Efstathiou and Hadjiioannou, 1995).

In contrast to strong oxyacids of halogens such as perchloric or perbromic acid, periodic acid (HIO) can be treated as a weak polyprotic acid in water and exists through dissociation in water, generating several protonated and deprotonated forms of the species (Efstathiou and Hadjiioannou, 1995). The oxidation potential, stability, and catalytic activity and electroactivity of PIT depend on the equilibrium between all the species formed. Orthoperiodic acid HIO is in dynamic equilibrium with tetrahydroxy periodate ion HIO, trihydroxy periodate ion HIO, and metaperiodate ion IO, and so on, along with the hydrolysis and dimerization processes in an alkaline condition (Equation 5) (Chen et al., 2022; Efstathiou and Hadjiioannou, 1995; Zhou et al., 2024). Therefore, the species-distribution is strongly pH-dependent and is only weakly influenced by ionic strength and total concentration.

$$H_{5}IO_{6}⇌H_{4}IO_{6}^{-}⇌H_{3}IO_{6}^{2-}.$$ (5)

In the extremely acidic solution (pH < 2), orthoperiodic acid is the predominant species in the solution, while during the course of deprotonation at the higher pH, HIO and HIO species will become dominant. It is in the pH region of approximately 2–8 that metaperiodate ion dominates as the main oxidizing agent (Figure 1) (Efstathiou and Hadjiioannou, 1995). In the very strongly alkaline solution, IO ion will be virtually absent due to its hydrolysis and complex formation with the build-up of the hydroxide ions. Under this condition ortho-periodate anions are formed as the polymeric or dimeric species such as HIO and the less active oxidizing agent (Figure 2) (Efstathiou and Hadjiioannou, 1995; Zhou et al., 2024). In view of this, the pH of the solution is important for maintaining both the stability of the PIT and the kinetics of the reaction, along with the effectiveness of the electroanalysis.

The oxidation power of PIT is controlled by both the predominant species and standard reduction potentials in the solution. Under extremely acidic conditions, PIT is a very strong oxidizing agent with the standard potential nearly up to +1.6V vs. SHE, and it readily oxidizes organic and inorganic species, such as alcohol, vicinal diols, carbohydrates, amino compounds, and transition-metal compounds (Equation 6) (Arndt et al., 2022a; Efstathiou and Hadjiioannou, 1995; Mecozzi et al., 2019; Wong and Shing, 2014). Meanwhile, under highly alkaline conditions, the standard potential is approximately +0.7V (Equation 7); this significant decrease in potential values shows that the oxidation power of PIT in alkaline conditions is low. This reduction is caused by the increasing amount of hydroxide ion and decreasing concentration of IO ions and less active complex species as the ortho-periodate ions. PIT oxidation reactions are favored under acidic or near-acidic conditions.

$$\text{Acidic solutions}:H_{5}IO_{6\;}+H^{+}+2e^{-}⇌\;IO_{3}^{-}+3H_{2}O\;\;E^{0}\approx +1.6\;V.$$ (6)

$$\text{Alkaline solutions}:H_{3}IO_{6}^{2-}+2e^{-}\;⇌\;3OH^{-}+IO_{3}^{-}\;E^{0}\approx +0.7\;V.$$ (7)

The pH-dependent characteristics of PIT can play a crucial role in synthetic chemistry, analytical chemistry, electrocatalysis, environmental applications, and medicinal chemistry. For instance, in PIT-mediated oxidation, acidic media are usually preferred for the maximum efficiency and reaction rate in oxidation, and it can be preferred under the mild activity condition in certain selective oxidation processes. For electrochemical sensing, pH plays a crucial role in the electron-transfer rate, peak current magnitude, sensor selectivity, catalytic performance, and electrode stability. It has been shown that some electrochemical PIT-based sensors present the maximum efficiency in certain pH ranges because of the control of redox mechanisms at the electrode surface by the dominant PIT species. There is a lack of comparative study based on the detailed and systematic relation among the PIT species, electrochemical performance, catalytic activity, and analytic sensitivity. This knowledge will provide guidance for designing new sensors, catalytic systems, and environmental applications.

### 2.3 Preparation methods of periodate species

Periodate species can be synthesized via several chemical and electrochemical routes with varying efficiency, selectivity, and environmental impacts. The earliest and most established method involves chemical oxidation of iodate under strongly alkaline conditions using chlorine gas, producing sodium hydrogen periodate (Na_3_H_2_IO_6_) as a sparingly soluble precipitate (Equation 8), which can be readily isolated with yields exceeding 80% (Willard and Ralston, 1932). However, large-scale applications are limited due to the toxicity and handling risks associated with chlorine gas. Subsequently, several alternative chemical oxidants such as hypochlorite, chlorates, hydrogen peroxide, ozone, and xenon-based compounds have been utilized for iodate or iodide oxidation to periodate species (Arndt et al., 2022a). Although effective, these approaches often suffer from low selectivity, formation of by-products, and difficult product separation, limiting their sustainability.

$${\text{IO}}_{3}^{-}+{\text{Cl}}_{2}+4{\text{OH}}^{-}+3{\text{Na}}^{+}\;\to \;{\text{Na}}_{3}H_{2}{\text{IO}}_{6}+2{\text{Cl}}^{-}+H_{2}O.$$ (8)

Electrochemical synthesis provides a more controllable and environmentally cleaner alternative for periodate production, enabling precise regulation of the key operational parameters such as the current density (j), electrolyte composition, pH, temperature, and electrode potential. These variables strongly influence the efficiency of iodine oxidation, product selectivity, and suppression of side reactions. Early electrochemical systems utilized platinum electrodes under alkaline conditions to oxidize iodide to periodate, demonstrating the feasibility of direct anodic conversion but with moderate efficiency and limited scalability (Willard and Ralston, 1932). Subsequent developments significantly improved the performance through the use of lead dioxide (Pb/PbO_2_) anodes, which offer higher overpotentials for oxygen evolution and enhanced oxidative capability toward iodide and iodate intermediates. Further optimization was achieved by introducing additives such as K_2_Cr_2_O_7_, which facilitates the formation of a protective chromium oxide layer on electrode surfaces. This layer suppresses the cathodic back-reduction processes and minimizes electrode fouling, thereby improving the Faradaic efficiency, product stability, and overall yield of periodate formation (Equation 9). Conditions: Pb/PbO_2_ anode, undivided cell, j = 150 mA cm^-2^–300 mA cm^-2^, KI (1.0 M–2.5 M), K_2_Cr_2_O_7_ (0.5 g L^-1^), and 50 °C–60 °C (Achanta et al., 2025; Kempler and Nielander, 2023). In addition, the Pb/PbO_2_ systems benefit from their chemical robustness in strongly oxidizing and acidic environments, enabling more stable long-term operation compared to that with noble metal electrodes, although concerns remain regarding lead toxicity and the environmental impact.

$$2\text{KI}+4H_{2}O\;\to \;2{\text{KIO}}_{4}+8H^{+}+8e^{-}.$$ (9)

In addition, divided (fractional) electrochemical cells were introduced to suppress undesired reverse reactions and improve current efficiency by physically separating anodic and cathodic processes, particularly under non-basic or mildly acidic conditions. This configuration minimizes the reduction of the generated periodate species at the cathode and enhances product stability. Significant improvement in performance was achieved using PbO_2_ electrodes, which exhibit high anodic overpotential for oxygen evolution and, thus, favor selective oxidation pathways (Equation 10). Their compatibility with acidic media further enhances the process flexibility, as the high potential required for oxygen reduction limits the parasitic side reactions and improves the overall faradaic efficiency (Arndt et al., 2022a).

$${\text{KIO}}_{3}\;⟶\;{\text{KIO}}_{4}.$$ (10)

Conditions: Pt, Pd, or PbO_2_ anode; undivided electrolysis cell; j = 10 mA cm^-2^–79 mA cm^-2^; KOH or H_2_SO_4_; K_2_Cr_2_O_7_ (0.1 wt%); 10 °C–86 °C.

As electrochemical technology advances, BDD electrodes have emerged as a next-generation material for periodate synthesis owing to their exceptional chemical inertness, mechanical robustness, wide electrochemical potential window, and strong resistance to surface fouling. In addition, BDD electrodes are non-toxic, free of metallic impurities, and exhibit stable performance under both strongly acidic and alkaline conditions, making them highly suitable for green and sustainable electrochemical processes (Arndt et al., 2020). These combined properties clearly distinguish BDD from conventional metal-based electrodes and enable more efficient and environmentally benign anodic oxidation systems. Furthermore, BDD-based systems support continuous production through flow reactor configurations, which minimizes precipitation issues, improves mass transport, and simplifies downstream product separation, while maintaining high operational durability.

**FIGURE 3:**
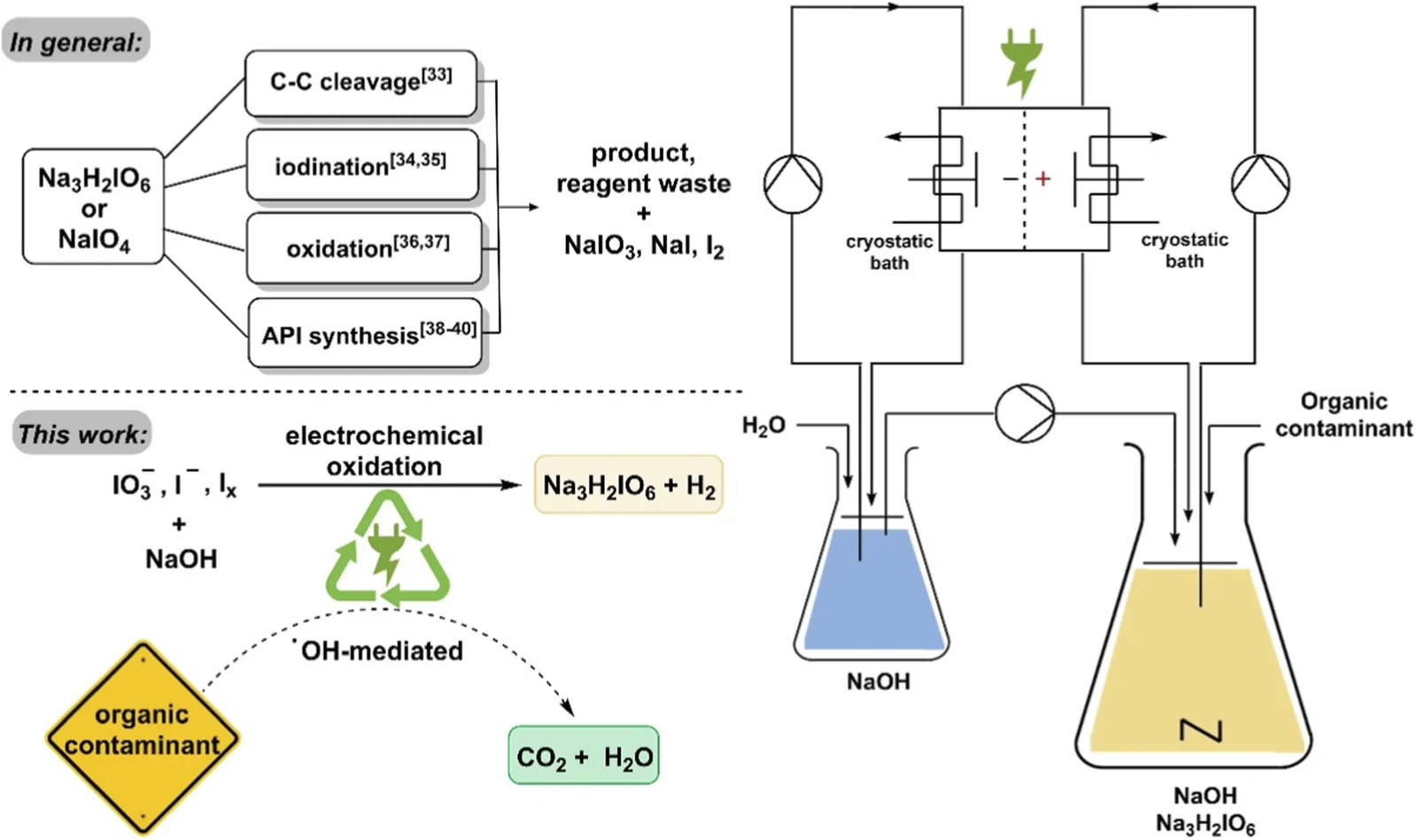
Self-cleaning electrochemical production and recycling of periodate at BDD anodes. (Left) Organic contaminants in iodate/periodate streams are mineralized to CO_2_ and H_2_O via hydroxyl radical-mediated “cold combustion,” while iodate is re-oxidized to periodate. The process tolerates dyes, pharmaceuticals, and iodinated compounds, enabling sustainable oxidant regeneration and upcycling to high-purity periodate. (Right) Schematic illustration of the platform process and cryostat pump setup. Adapted from Kisukuri et al. (2022) (Open access, 2022).

Kisukuri et al. (2022) demonstrated a robust and self-cleaning electrochemical strategy for the production and recycling of PIT using BDD anodes (Figure 3). The study showed that trace organic contaminants present in recovered iodate or spent periodate solutions do not hinder the electrochemical re-oxidation to PIT. Instead, under hydroxyl radical-mediated conditions, organic impurities are mineralized to CO_2_ and H_2_O through a “cold combustion” process, enabling the simultaneous purification and oxidant regeneration. Various dyes, pharmaceuticals, and iodine-containing compounds were analyzed as model contaminants. Product formation and contaminant degradation were monitored by UV–Vis spectroscopic techniques (NMR, MS, and LC–MS). As a representative example, dimethyl 5-iodoisophthalate (2 mM) was degraded by 90%, 95%, and 99% while generating 0.042, 0.054, and 0.082 kilo-equivalents of PIT, respectively. The approach also enabled upcycling of the iodine-containing waste streams, including the contrast media, into high-purity PIT, highlighting its potential as a sustainable and circular platform of oxidation process.

**FIGURE 4:**
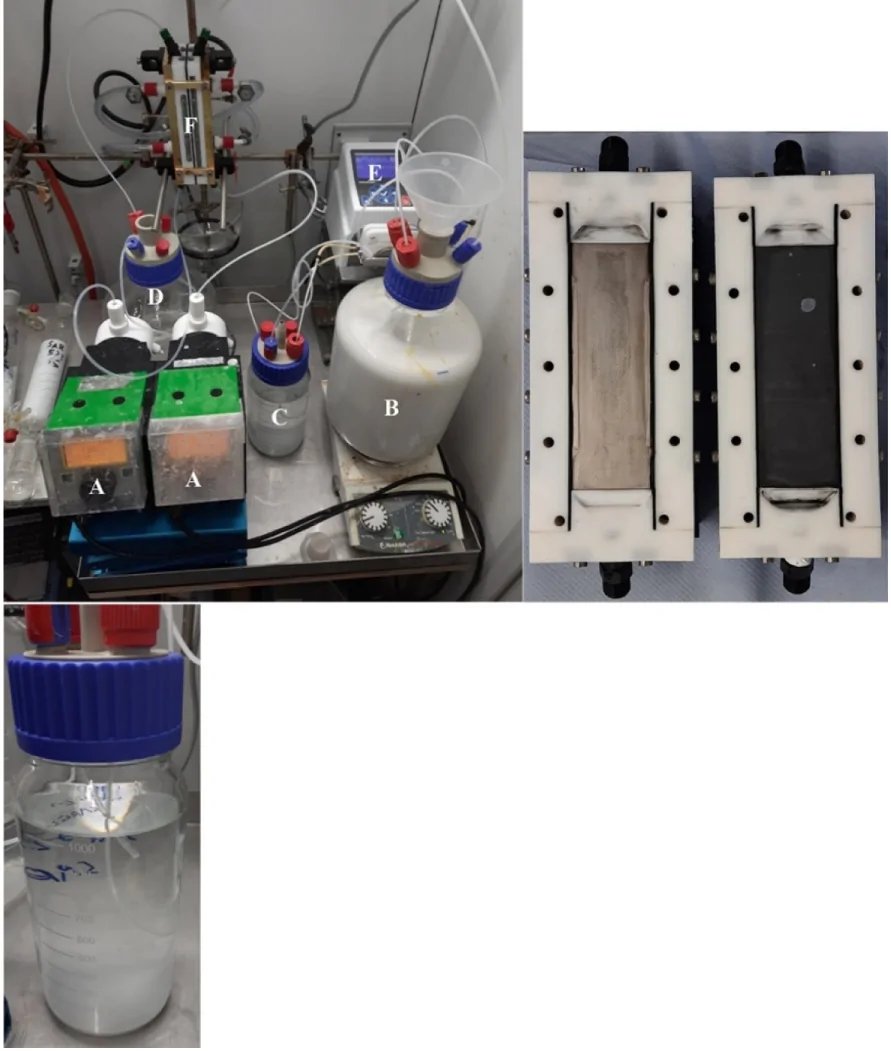
Continuous-flow electrochemical reactor for the direct synthesis of sodium periodate using cyclic-flow electrolysis and an e-CSTR system under alkaline conditions (Arndt et al., 2022b). The steady-state setup includes two membrane pumps **(A)**, a stirred anolyte reservoir **(B)**, inline product separation **(C)**, a catholyte reservoir **(D)**, a dual-channel peristaltic pump **(E)**, and a flow electrolyzer **(F)**, enabling continuous operation and electrolyte management; the figure also shows the disassembled electrolysis cell and a magnified view of the sequestration vessel.

Recently, Arndt et al. (2022b) developed a continuous-flow electrochemical reactor integrating cyclic flow electrolysis with a continuously stirred tank reactor (e-CSTR) to enable steady-state, fully continuous production of sodium periodate from iodide under strongly alkaline conditions (Figure 4). The system is designed for scalable and cost-efficient synthesis through flexible feedstock handling, precise control of reaction parameters, inline product separation, and efficient electrolyte recycling. This hybrid configuration overcomes the limitations of batch processing by enhancing mass transport, improving energy efficiency, and enabling long-term operational stability. As shown in Figure 4, the setup incorporates membrane pumps, stirred reservoirs, and a flow electrolyzer, which collectively ensure controlled reagent circulation and continuous oxidant generation.

Alternative routes have been explored for the direct electrochemical oxidation of iodide to periodate via an eight-electron-transfer process; however, these approaches are frequently constrained by the precipitation of intermediate iodine species, limited solubility of high-valent intermediates, and competing side reactions, which collectively complicate product isolation and often necessitate multi-step iodide–iodate–periodate conversion pathways (Arndt et al., 2022a; Efstathiou and Hadjiioannou, 1995). In a representative system, NaI (0.4 M) is oxidized using a boron-doped diamond (BDD) anode at j = 100 mA cm^-2^ in a divided cell equipped with a Nafion membrane to suppress cathodic reduction in 4 M NaOH at room temperature, yielding Na_3_H_2_IO_6_ as the final product (Equations 11–15). The anodic process proceeds through step-wise electron transfer and hydroxide-assisted oxidation of iodide to periodate, while the cathodic reaction involves water reduction with hydrogen evolution and the regeneration of hydroxide ions. Under strongly alkaline conditions (pH = 10), additional acid–base equilibria govern the formation of hydrogen periodate species, reflecting the complex speciation behavior of periodate in solutions. Despite these advances, long-standing challenges in the selectivity, stability, and solubility remain the central limitations in periodate synthesis, even in modern electrochemical systems (Arndt et al., 2022a).

$$\text{NaI}+\text{aq}.\text{ NaOH }\left(4\;M\right)\;⟶\;Na_{3}H_{2}IO_{6}.$$ (11)

Conditions: BDD anode; divided cell (Nafion membrane); j = 100 mA cm^-2^.

$$\text{Anode}:I^{-}+8\;OH^{-}\;\to \;IO_{4}^{-}+4H_{2}O+8\;e^{-}.$$ (12)

$$\text{Cathode}:8H_{2}O+8\;e^{-}\to 4H_{2}+8OH^{-}.$$ (13)

$$\text{Overall reaction}:I^{-}+4H_{2}O\;\to \;IO_{4}^{-}+4H_{2}.$$ (14)

$$\text{pH }10:IO_{4}^{-}+2OH^{-}\to \;H_{2}IO_{6}^{-3}.$$ (15)

## 3 Periodate for environmental sustainability and green chemistry

PITs are widely used in green chemistry due to their strong oxidizing ability, mild reaction conditions, and compatibility with eco-friendly electrochemical regeneration. Their application reduces the need for toxic heavy metals, minimizes waste generation, and enables selective organic transformations that are suitable for sustainable fine chemical production and flow chemistry processes (Arndt et al., 2022a). The main activation routes and applications are summarized in Table 2.

**TABLE 2:** Periodate-based methods and their mechanisms in green chemistry and environmental sustainability applications.

Method/Activation system	Role and mechanism of periodate	Applications/Target pollutants	Ref.
Direct periodate oxidation	Electrophilic oxidation and oxygen transfer under mild conditions	Alcohols, phenols, dyes, and pharmaceuticals	Arndt et al. (2022a), Sklarz (1967), Sudalai et al. (2015), Zhou et al. (2024)
Malaprade reaction (vicinal diol cleavage)	Cyclic periodate ester formation followed by C–C bond cleavage	Sugars, polyols, and biomass derivatives	Arndt et al. (2022a), Dyer (1965), Sklarz (1967), Sudalai et al. (2015), Zhong and Shing (1997), Zhou et al. (2024)
Radical-based AOP (chemical activation)	Generation of reactive oxygen/iodine species (•OH, IO•, and O•)	Antibiotics, dyes, pesticides, and APIs	Arndt et al. (2022a), Chia et al. (2004), Dyer (1965), Ermolinsky and Mikhailov (2000), Merouani and Hamdaoui (2021)
Metal-catalyzed activation (Fe, Mn, Ru, and Os)	Metal-mediated electron transfer enhancing IO_4_^−^ activation	Refractory organics and chlorinated compounds	Arndt et al. (2022a), Chen et al. (2022), Chia et al. (2004)
Electrochemical activation	Anodic generation/regeneration of periodate (BDD, PbO_2_, and carbon electrodes)	Wastewater contaminants and pharmaceuticals	Sukhatskiy et al. (2023)
Photo-assisted activation (UV/light)	Photolysis of IO_4_^−^ producing reactive radical species	Dyes, phenols, and chemical agents	Merouani and Hamdaoui (2021), Sukhatskiy et al. (2023), and Zhang et al. (2024)
Bio-electrochemical activation	Electron transfer from microbes generating ROS and activating IO_4_^−^	Carbamazepine and micropollutants	Zou et al. (2024)
Nanoparticle/ultrasound-assisted systems	Surface catalysis and cavitation enhance radical production	Phenol and aromatic pollutants	Darvishi Cheshmeh Soltani and Safari (2016)
Biomass and polymer oxidation	Selective oxidation of biopolymers to functional materials	Cellulose, chitosan, and alginates	Chimpibul et al. (2020), Liu et al. (2020), Merouani and Hamdaoui (2021), Nypelö et al. (2021)
API synthesis and green transformations	Periodate-assisted oxidation and Lemieux–Johnson reactions	Pharmaceutical intermediates and APIs	Arndt et al. (2022a), Dyer (1965), Ermolinsky and Mikhailov (2000)

PIT has been extensively analyzed as an environmentally benign oxidant in AOPs for water and industrial wastewater treatment. It is effective for degrading a wide range of organic pollutants, including alcohols, phenols, dyes (e.g., Reactive Black 5, methylene blue, acid blue 25, and alizarin violet N), pesticides, pharmaceuticals, and active pharmaceutical ingredients (APIs), often with minimal changes in the pH or solution appearance (Lee and Yoon, 2004; Parent et al., 2012; Sklarz, 1967; Wong and Shing, 2014; Zhang et al., 2024; Zhou et al., 2024). In addition, PIT plays an important role in both the synthesis and degradation of APIs, which are major contributors to industrial pollution due to their large-scale production (Munarin et al., 2012; Yang et al., 2023; Clamp and Hough, 1966).

PIT-based AOPs operate through radical oxidation, electron transfer, and high-valent metal-mediated pathways, enabling efficient degradation of diverse pollutants. These systems are simpler to operate and consume less energy and chemicals than conventional processes while achieving simultaneous oxidation and disinfection, thus reducing the operational cost and improving sustainability (Chimpibul et al., 2020; Merouani and Hamdaoui, 2021; Sklarz, 1967; Clamp and Hough, 1966). Mechanistically, reactions proceed via electrophilic addition, nucleophilic attack, oxygen atom transfer, hydride transfer, hydrogen atom transfer, and electron transfer pathways, which are all driven by the high-valent iodine species (Chimpibul et al., 2020; Merouani and Hamdaoui, 2021; Yang et al., 2023; Clamp and Hough, 1966; Zhang et al., 2024).

**FIGURE 5:**
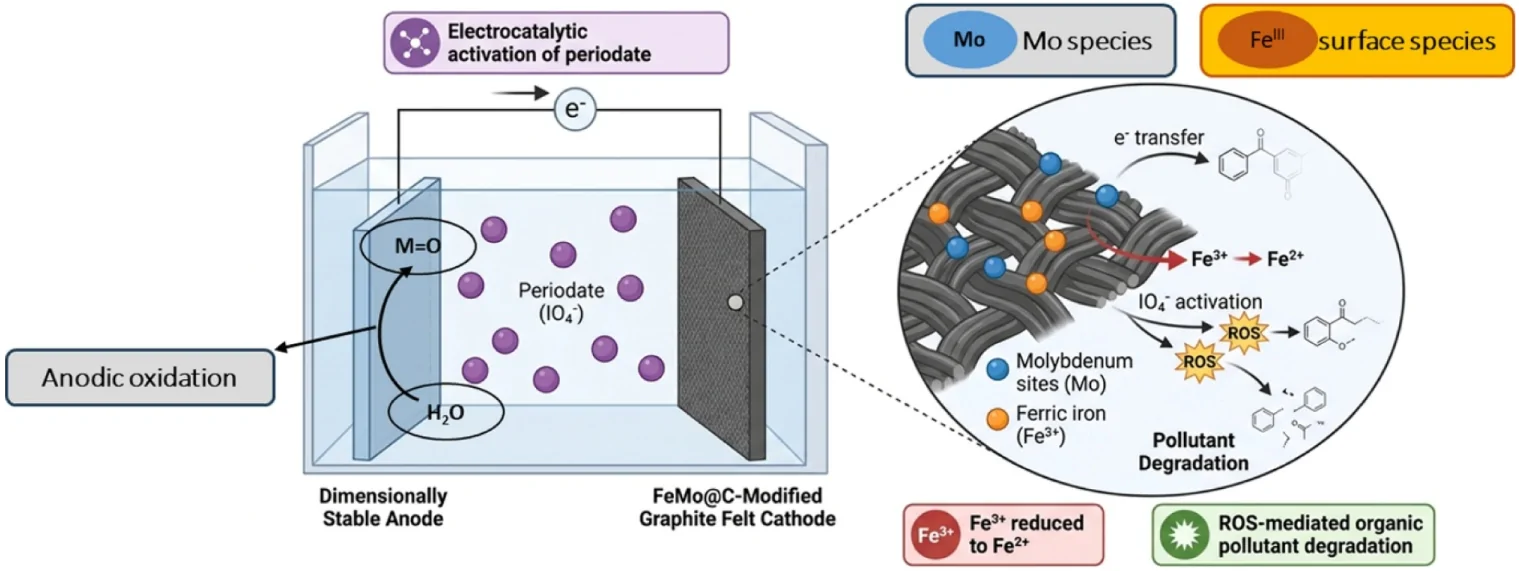
Synergistic electrochemical and bimetallic FeMo@C activation of periodate for contaminant degradation. Electron-rich Mo(IV) sites enhance electron transfer and promote the Fe(III)/Fe(II) redox cycle, enabling efficient generation of reactive species, with iodate radicals (IO_3_•^-^) identified as dominant. The coupled electro-catalytic system achieves markedly improved degradation kinetics than individual electrochemical or catalytic processes and remains effective in real water matrices. Adapted from Ganiyu et al. (2026), Elsevier.

Recent advances have focused on enhancing PIT activation using electrochemical, photochemical, and bio-assisted systems. Electro-bioactivation strategies generate reactive oxygen species (e.g., IO•, OH•, and O•) through interactions between electroactive bacteria and periodate on electrode surfaces, enabling rapid degradation of carbamazepine, including the complete removal under acidic conditions and efficient treatment of real wastewater without external voltage (Chimpibul et al., 2020; Merouani and Hamdaoui, 2021; Sklarz, 1967; Wong and Shing, 2014; Yang et al., 2023; Clamp and Hough, 1966; Zhou et al., 2024; Zou et al., 2024). Photoactivated systems using monochromatic light or 222 nm or 254 nm UV irradiation enable the degradation of 4-chlorophenol, chemical warfare hydrolyzylates, and Reactive Black 5, respectively (Chia et al., 2004; Tang and Weavers, 2007; Tang and Weavers, 2008). A broad spectrum of pollutants, including quinoline, benzoic acid, bisphenol A, nitrobenzene, sulfamethoxazole, trichlorophenols, perfluorooctanoic acid, methylene blue, and other aromatic compounds, has also been efficiently degraded, with the performance strongly dependent on the PIT concentration, pH, light intensity, nanoparticles, and temperature. These activation effects are illustrated in Figure 5, highlighting the nanoparticle- and ultrasound-assisted phenol degradation.

Beyond pollutant removal, PIT contributes to the degradation of bio-refractory contaminants such as endocrine-disrupting chemicals, pharmaceuticals, and dyes (Sukhatskiy et al., 2023). It also enables oxidation of biopolymers including cellulose, alginates, chitosan, and hyaluronan, thus producing functional materials such as cellulose nanofibrils and chitin microcrystals for wastewater treatment applications (Arndt et al., 2022a; Liu et al., 2020; Nypelö et al., 2021; Sklarz, 1967). In addition, PIT mediates controlled oxidation of nucleotides and nucleic acids, yielding modified nucleosides with valuable biochemical properties (Ermolinsky and Mikhailov, 2000a). In most systems, C–H bond activation occurs first, followed by oxidation, bond cleavage, and molecular fragmentation (Arndt et al., 2022a; Mecozzi et al., 2019; Sudalai et al., 2015; Wong and Shing, 2014; Zhou et al., 2024).

Ganiyu et al. (2026) developed a synergistic electrochemical–catalytic system for PIT activation using a bimetallic FeMo@C-coated graphite felt cathode (Figure 5). The system enables efficient degradation of contaminants (e.g., bisphenol A) through coupled electro- and co-catalytic processes. Characterization revealed that electron-rich Mo(IV) sites enhance electron transfer and promote the Fe(III)/Fe(II) redox cycle, thus significantly improving the catalytic performance. The integrated system achieved a high degradation rate (k-app = 0.35 min^-1^) with a strong synergy index of 9.3, outperforming electrochemical or catalytic processes alone. Mechanistic studies (EPR, quenching, and probe tests) identified iodate radicals (IO_3_•^-^) as the dominant reactive species, with minimal contribution from •OH radicals. The system also showed robust performance in real water matrices, demonstrating strong potential for practical wastewater treatment applications.

**FIGURE 6:**
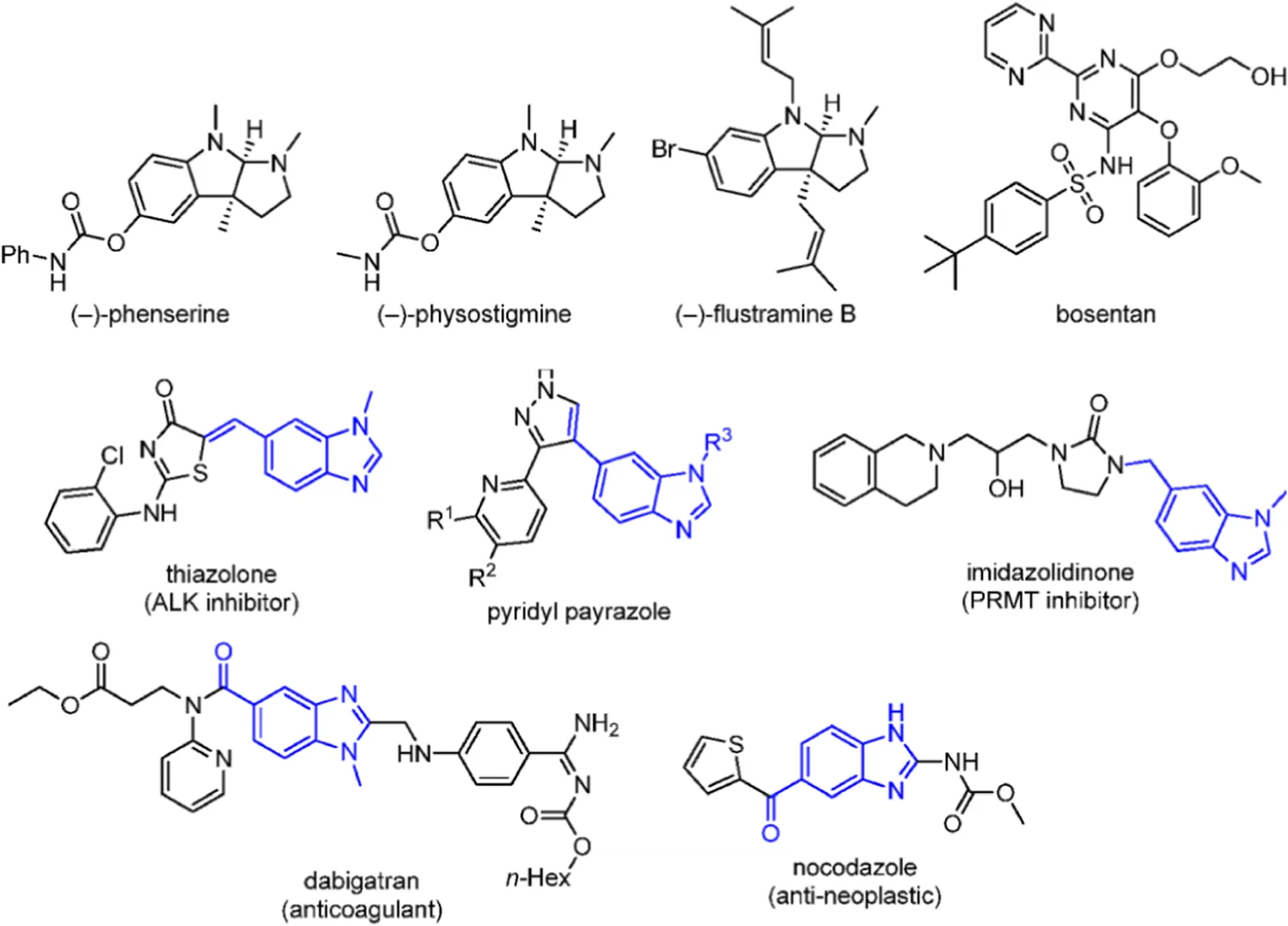
Chemical structures of representative active pharmaceutical ingredients synthesized via periodate-mediated transformations and benzimidazole derivatives.

From a green synthesis perspective, PIT is widely used in the preparation of active pharmaceutical ingredients such as bosentan, flustramine B, physostigmine, and phenserine (Arndt et al., 2022a). It is also applied in Lemieux–Johnson oxidation using Ru-, Os-, and RuCl-based catalysts in aqueous and organic solvents such as ethyl acetate, ammonium acetate, and dioxane (Arndt et al., 2022a; Osako et al., 2006; Plietker, 2005). PIT significantly improves industrial pharmaceutical synthesis by reducing the cost, reaction time, waste generation, and impurity formation, thereby enhancing environmental sustainability, as illustrated in Figure 6.

## 4 Analytical methods using periodate oxidation chemistry

Given the importance of PIT in analytical applications, the development of PIT-selective electrodes is of wide interest to researchers, particularly because of the difficulties of the response rate or sensitivity, detection limit, linear dynamic range (LDR), and selectivity (Aziz and Kamel, 2010; Chatraei and Zare, 2013). Electrochemical methods are among the most promising in terms of simplicity, speed, and rapid response to trace levels (Abdel-Haleem, 2016; Abdel-Haleem and Zahran, 2019; Ahmed et al., 2022; El-Beshlawy et al., 2022). To enhance selectivity and reduce the voltage required for electron transfer, strategies are currently being studied to improve the analytical performance by modifying the electrode surfaces with nanoparticles or complex molecules (Chatraei and Zare, 2013). PIT possesses a certain set of characteristics that make it an efficient reagent for chemical analysis, particularly because of its strong oxidation power enabling it to react with a wide range of organic and inorganic compounds under mild conditions of room temperature and pH. These applications exploit selective oxidation reactions and electrochemical behavior that can be applied in certain analytical determinations (Aziz and Kamel, 2010; Efstathiou and Hadjiioannou, 1995).

Malaprade reactions are among the more well-known PIT reactions that were used extensively for the quantitative analysis of the different organic analytes (Table 3); the reaction is used to split carbon–carbon bonds between two atoms bearing adjacent hydroxyl groups (vicinal diols) or similar bonds (Equation 16). In this reaction, PIT is oxidized to iodate (IO^−^_3_), and the amount of the organic reactant can be assayed by monitoring the PIT consumption determined by iodometry (Efstathiou and Hadjiioannou, 1995), and the remaining PIT can be titrated using sodium iodide (NaI) solution in an acidic medium (Equation 17), which releases molecular iodine, which is then titrated with sodium thiosulfate; alternatively, PIT can be monitored by assaying the products such as iodate, along with other compounds that can be assayed using this method, such as glycerol (Luca et al., 1998), sugars (Diamandis et al., 1983), and hydroxylated amino acids (Zayed et al., 1986). This method is one of the most accurate classical methods for the determination of trace amounts of compounds containing oxidizable groups.

$$R\left(OH\right)C-C\left(OH\right)R+IO_{4}^{-}\to R\left(=O\right)C+C\left(=O\right)R+IO_{3}^{-}+H_{2}O.$$ (16)

$$IO_{4}^{-}+8I^{-}+8H^{+}\to 9I_{2}+4H_{2}O.$$ (17)

**TABLE 3:** Analytical applications of PIT oxidation chemistry based on Malaprade-type reactions and electrochemical detection strategies.

Application	Target analyte	Chemical principle (PIT reaction)	Ref
Vicinal diol analysis (Malaprade reaction)	Organic compounds containing adjacent hydroxyl groups	Periodate oxidizes vicinal diols, cleaving carbon–carbon bonds and forming carbonyl compounds with the reduction of IO_4_^−^ to IO_3_^−^	Chimpibul et al. (2020) and Efstathiou et al. (2002)
Sugar analysis	Carbohydrates and sugar derivatives	Oxidative cleavage of carbon–carbon bonds between hydroxylated carbons under mild aqueous conditions	Diamandis et al. (1983)
Glycerol determination	Glycerol and other polyhydric alcohols	Periodate oxidation of triols producing carbonyl compounds, quantified via iodometric back titration	Luca et al. (1998)
Amines and binary amines	Organic amine derivatives	Oxidative transformation of amine-containing compounds under periodate conditions enabling indirect quantification	Zayed et al. (1986)
Hydroxylated amino acids	Serine, threonine, and related compounds	Periodate oxidation of hydroxyl-functional amino-acid side chains producing measurable redox products	Martínez-Lozano et al. (1991) and Zayed et al. (1986)
Electrochemical sensing (PIT-selective electrodes)	Trace organic and inorganic analytes	Redox interaction of periodate with modified electrode surfaces enhanced by nanoparticles or complex ligands	Darvishi Cheshmeh Soltani and Safari (2016) and Plietker (2005)

## 5 Electrochemical methods for periodate detection

Electrochemical techniques are widely used for periodate determination due to their high sensitivity, rapid response, low cost, and suitability for real-time analysis (Abdel-Haleem et al., 2016; Abdel-Haleem et al., 2024; El-Beshlawy et al., 2024). Despite the irreversible electrochemical behavior of PIT, reliable quantification has been achieved using potentiometry, amperometry, and voltammetry. Potentiometric methods typically utilize PIT-selective membrane electrodes, where the potential response correlates with the PIT concentration. Amperometric approaches monitor current changes linked to PIT-induced redox processes, which are often enhanced by modified electrode surfaces. Voltammetric techniques, including cyclic and differential pulse voltammetry, provide improved sensitivity through catalytic or indirect PIT signals. Nanomaterial-modified and ion-selective electrodes further enhance the selectivity, stability, and detection performance for environmental and catalytic applications (Aziz and Kamel, 2010; Guerreir et al., 2010; Guo et al., 2008; Luca et al., 1998).

### 5.1 Potentiometric methods for periodate detection

Potentiometric determination of PIT is based on measuring the potential difference across a selective membrane that responds to PIT activity in a solution. The generated potential follows ion-selective electrode principles and is proportional to the logarithm of PIT concentration according to phase-boundary ion-exchange equilibria. Various potentiometric sensors for PIT and related species have been reported (Table 4), offering simple instrumentation, rapid response, low cost, portability, and suitability for continuous monitoring and flow injection analysis (FIA). The most reported PIT-selective electrodes are based on polymeric membrane systems incorporating ion-pair complexes as electroactive materials, where selectivity arises from ion-exchange and partitioning processes within the membrane phase. However, ionophore-based membranes, which rely on specific molecular recognition, remain underexplored despite their potential advantages in improving the selectivity, stability, and resistance to interference in complex matrices.

**TABLE 4:** Potentiometric sensing strategies for PIT and related analytes, including the electrode systems, analytical performance parameters, and response characteristics.

Analyte	Electrode	Conc. range	Slope (mV/Decade)	pH	Response time	Ref.
Ascorbic acid	Ni(II)-Schiff base	2 µg/mL–13 µg/mL	4.97	3–8	<10 s	Aziz and Kamel (2010)
PIT	PVC/Tetraphenylphosphonium	1.5 × 10^−6^ M–1 × 10^−1^ M	52.5	3–10	5 s–15 s	Matter and Ayad (2022)
IO_4_^−^	Cetylpyridinium chloride/PVC	10^−5^ M–10^−2^ M	58.1	2–9	30 s–40 s	Othman (2010)
Dopamine	Tubular flow electrode/PVC	8.0 × 10^−3^ g/L–2.7 × 10^−1^ g/L	310.1	-	-	Conceiçäo et al. (1993)
Glucose	PIT-flow system	50 mg/100 mL–290 mg/100 mL	-	-	-	Diamandis et al. (1983)
Sucrose	PIT-flow system	3 mM–18 mM	-	-	-	Diamandis et al. (1983)
Sucrose	ISE	0.01 M–0.1 M	-	-	-	Hartofylax et al. (1989)
Glycerol	Single-FIA ISE	20 mg/L–500 mg/L	-	-	-	Gervasio et al. (2001)
Glycerol	Kinetic-ISE	0.56 µM–24 µM	-	4.5	<2 min	Hartofylax and Efstathiou (1985)
Glycerol	FIA-TOA	1,000 mg/L–5,000 mg/L	-	-	-	Conceiçäo et al. (1993)
Glycerol/2,3-butanediol	PVC-Tetraphenylphosphonium PIT	-	-	-	-	Conceiçäo et al. (1993)
α-diols/carbohydrates/hydrazine	Nitron-PIT/nitrobenzene	0.05 mg/mL–2.5 mg/mL	55	3–7	<1 min	Massan and Elsaied (1987)
PIT	Berb-PIT/o-nitrotoluene	10^−1^ - 5 × 10^−5^ M	62	1.5–7.5	5 s–10 s	Jain et al. (1989)
PIT	Caproquat-PIT	10^−1^ M–10^−7^ M	60	2.5–7.5	-	Kudoh et al. (1980)

**FIGURE 7:**
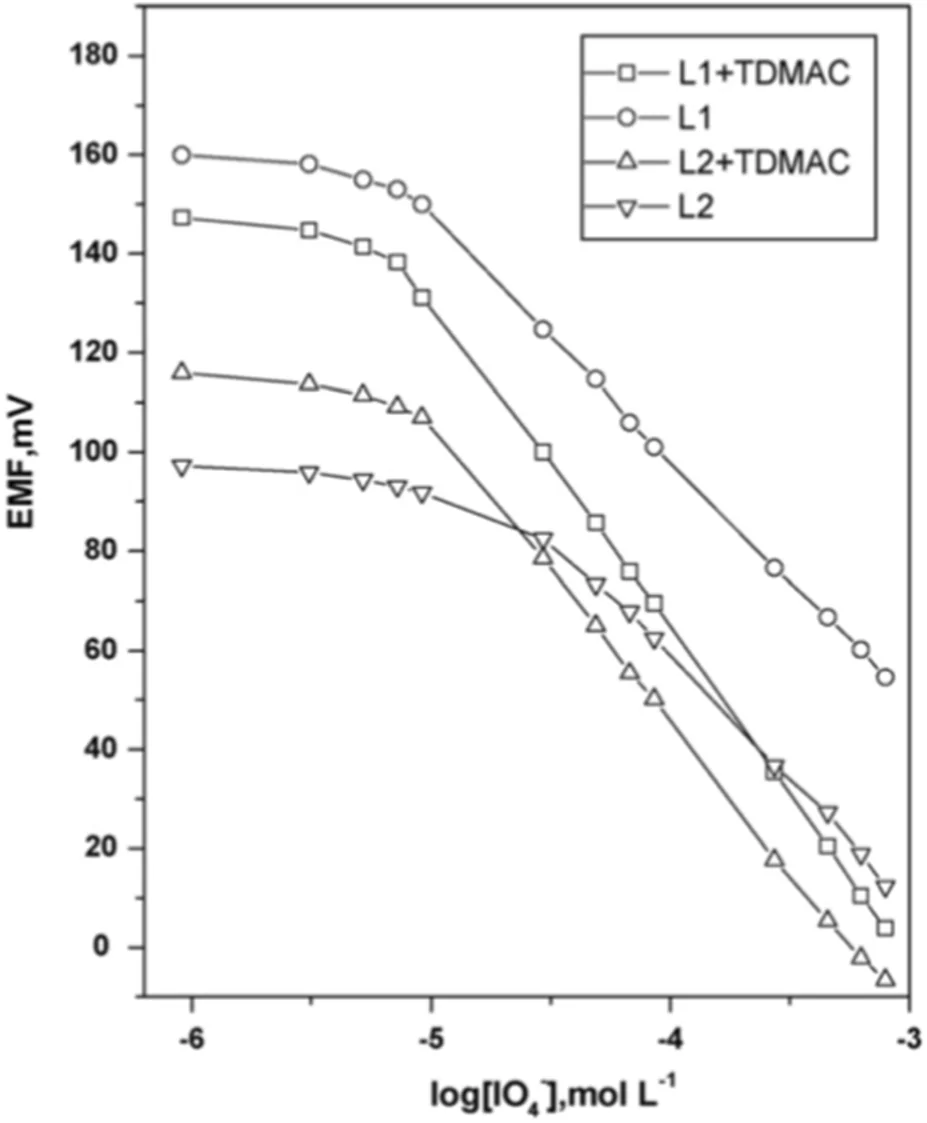
Potentiometric response of ionophore-based periodate-selective membrane sensors showing the relationship between the electromotive force (EMF) and periodate concentration under optimized measurement conditions (Aziz and Kamel, 2010).

Aziz and Kamel (2010) developed an ionophore-based periodate-selective electrode using a neutral carrier mechanism (Figure 7). The sensing membrane incorporated nickel(II)-N,N-bis(salicylaldehyde)-4,5-dimethyl-1,2-phenylenediamine (L) and its Ni(II)-complex to construct two sensors for the potentiometric detection of periodate under static and hydrodynamic conditions. This work is notable as the first reported PIT-selective electrode based on an ionophore mechanism rather than conventional ion-exchange, providing enhanced selectivity and specificity toward periodate ions. Under static conditions, the sensors exhibited slopes of 66.1 and 59.9 mV/decade with detection limits of 5.2 × 10^−6^ and 7.3 × 10^−6^ M, respectively. In the hydrodynamic FIA mode, a slope of 71.1 mV/decade, detection limit of 7.3 × 10^−6^ M, and linear range of 1.0 × 10^-3^–1.0 × 10^−5^ M were achieved. The response time was below 10 s over a pH range of 3–8. The tubular sensor was further applied for the indirect determination of ascorbic acid in beverages and pharmaceuticals, achieving a linear range of 2 µg/mL–13 µg/mL, recovery of 98.8%, RSD of 1.3%, and throughput of up to 50 samples per hour without sample pre-treatment (Aziz and Kamel, 2010).

Matter and Ayad (2022) prepared the PIT-selective electrode with a polyvinyl chloride (PVC) membrane that used 2,3,5-triphenyltetrazolium-periodate (TT-IO), dioctyl phthalate (DOP) plasticizer, and sodium tetraphenylborate (NaTPB) as an ion exchanger. A Nernstian response of 50 mV/decade–55 mV/decade in concentration ranging from 1.5 × 10^−6^ M to 1.0 × 10^−1^ M was observed with rapid response time of 15 s and operational lifetime of approximately 90 days. The sensors were also reported to be insensitive to pH changes within the range 3–10 and showed high selectivity in the presence of other interfering ions. The method was used to determine PIT in well-water and waste-water samples from Awjila and Jalu regions of Libya (Matter and Ayad, 2022). This method is considered a good method in terms of the long lifetime of the sensor, in addition to the fast response time, which facilitates real-time and in-field monitoring.

A plasticized membrane sensor based on periodate–cetylpyridinium ion complex was also reported to measure PIT (Othman, 2010). The sensor provided anionic Nernstian response with a stable slope of 58.1 mV/decade in a concentration range of 10^−5^ to 10^−2^ M with a detection limit of 2.0x10^−6^ M. The response time of the electrode was 40 s, and stable responses were obtained over a wide pH range of 2–9. The sensor was utilized to determine PIT directly and to analyze various analytes, including hydrazine derivatives, table salt, and aminophenol derivatives, among others, indirectly with satisfactory accuracy (Othman, 2010). Although the sensor is based on the traditional ion-exchanger mechanism, it was applied successfully for real samples with reliable results.

Dopamine determination in pharmaceutical formulations was performed through oxidation with PIT by using a flow injection system that incorporated a tubular electrode with no internal reference solution that had a PVC membrane containing bis(triphenylphosphoranylidene) ammonium ion exchanger and 2-nitrophenyloctylether as plasticizer. A linear concentration range of 8.0 × 10^−3^ g/L–2.7 × 10^−1^ g/L was obtained with repeatability of 0.4 mV. Approximately 200 samples/hours were measured with no sample pre-treatment, and the result was in agreement with the USP method (Montenegro and Sales, 2000). The method is advantageous in terms of the wide concentration range.

Another PIT-selective liquid membrane electrode using a berberine–periodate ionic complex dissolved in o-nitrotoluene was reported (Jain et al., 1989). The sensor exhibited a nearly Nernstian response within the concentration range of 10^−1^–5.0 x 10^−5^ M and a response slope of 62 mV/decade; the response time was lower than 10 s and pH-dependent from 1.5 to 7.5. The detection limit was 2.5 x 10^−5^ M. The electrode was sensitive to PIT directly even in the presence of interference of iodate and bromate (Jain et al., 1989). A liquid membrane electrode of PIT was based on an ion pair formed between PIT and Capriquat extract into a nitrobenzene membrane. The electrode had a Nernstian response of 60 mV/decade over a concentration range of 10^−1^ M–10^−7^ M. Selectivity coefficients toward several ions were analyzed, and a stable working behavior was observed in the pH range 2.5–7.5. The sensor was successfully used in the potentiometric titration of di-alcohols and amino alcohol compounds (Kudoh et al., 1980).

Most of the reported PIT-selective electrodes, apart from the ionophore-based sensor of Aziz and Kamel (2010), were mainly based on the ion-pair recognition mechanism and not on specific coordination chemistry. This might be due to the irreversible electrochemical characteristic of the IO/IO redox couple leading to complexity on the direct reversible electrochemical sensing processes (Efstathiou and Hadjiioannou, 1995). Although ion-pair based sensors are more stable and sensitive in terms of potential response and simple, limited selectivity, the loss of membrane matrix and lower operational stability are shortcomings in comparison to ionophore-based recognition systems. Thus, the development of new PIT-selective ionophore, nanostructured membrane, and solid-contact potentiometric sensors is important for PIT electroanalysis.

Several reports have indicated the indirect determination of other organic compounds through PIT-mediated oxidation reactions using PIT-selective electrodes. Diamandis et al. (1983) developed a continuous-flow method using a PIT-selective electrode to determine reducing sugar in serum. The sample was introduced with a PIT excess in a flow system, and the residual PIT activity was measured by potential value. Linearity within 50 mg/100 mL–290 mg/100 mL was achieved with RSD 1%–2%. The method is highly appreciated for routine analysis, as approximately 30 samples/hour were successfully accurately and precisely measured by fully automated analysis without any pre-treatment (Diamandis et al., 1983). The method is of low sensitivity and selectivity.

A continuous-flow procedure was developed by Diamandis et al. (1983) to measure reducing sugars and sucrose mixture found in both natural and synthetic products. PIT was used in excess to react with reducing sugars. The remaining PIT concentration was measured using a PIT-selective sensor and linear correlation between the response, and the reducing sugar concentration was obtained over the range 3 mM–18 mM. Sucrose concentration was then calculated by subtracting from each other. Up to 30 samples/hours were automatically analyzed (Diamandis et al., 1983).

Indirect determination of sucrose and glycerol were also determined by measuring the residual PIT concentration after the reaction between PIT and sucrose/glycerol. A fast-response tubular electrode that integrated into a multi-site FIA system determined sucrose and glycerol simultaneously and reduced the analysis time by approximately 85% and the consumption of 190 g NaIO per analysis compared to that with traditional methods. Serial monitoring measurement of sucrose in sugarcane juice and syrup was achieved at approximately 50 measurements/hour with RSD values less than 2% (Gomez Neto et al., 1994). The method is advantageous in terms of reducing the use of chemicals, reducing the analysis time, and the simultaneous determination of glycerol and sucrose with high selectivity in very small time.

Hartofylax et al. (1989) presented the determination of sucrose by kinetic study on the rate of PIT reaction using a PIT-selective electrode at 0.01 M–0.10 M concentration range with average error of 1.4%. The method was successfully applied to dairy products and soft drinks (Hartofylax et al., 1989). An FIA system coupled with a PIT-selective electrode to determine glycerol was developed by Gervasio et al. (2001) through oxidation with PIT. A linear response was reported in the range 20 mg/L–500 mg/L, with recoveries of 96%–120% and the analysis rate of approximately 30 samples/hour (Gervasio et al., 2001). The selectivity is not high for this method.

PIT-selective electrodes have been utilized for kinetic glycerol determination, as demonstrated by Hartofylax and Efstathiou (1985), offering a linear response over 0.56 µM–24 µM with rapid analysis within 2 min at pH 4.5; the same system was further adapted for indirect alkaline phosphatase activity via p-glycerophosphate hydrolysis. Conceiçäo et al. (1993) developed a flow injection analysis (FIA) system based on PIT-selective electrodes without an internal reference solution using a tetraoctylammonium–periodate ion-exchanger membrane plasticized with 2-nitrophenyloctyl ether or dibutyl phthalate, achieving 40 samples h^-1^, high precision (RSD <0.005), and a glycerol range of 1,000 mg L^-1^–5,000 mg L^-1^. Additionally, a PVC membrane electrode incorporating bis(triphenylphosphoranylidene) ammonium periodate and 2-nitrophenyloctyl ether enabled the determination of glycerol and 2,3-butanediol in wine with recoveries of 96.0%–106.9% and a throughput of 33 samples h^-1^ using only 9.4 mg PIT per analysis (Luca et al., 1998). However, these systems exhibit limited selectivity due to PIT reactivity toward vicinal diols.

The determination of ascorbic acid in beverages and pharmaceuticals via PIT-mediated oxidation (Equation 18) was reported by Guerreir et al. (2010) using a PIT-selective PVC membrane potentiometric sensor incorporating a porphyrin ionophore and membrane additives. The sensor exhibited a slope of 73.9 ± 0.9 mV/decade over a PIT concentration range of 1.0 × 10^−6^ M to 6.0 × 10^−6^ M (17.7 µg/mL–194.0 µg/mL), with repeatability of 1.1 mV and an analytical throughput of 96 samples per hour. However, the method is limited by a narrow working concentration range, while electrode fouling due to reaction products and interference effects represents a key drawback.

$${IO}_{4}^{-}+{IO}_{3}^{-}.$$ (18)

Massan and Elsaied (1987) designed a PIT-selective liquid membrane electrode based on a nitrobenzene solution containing nitron-periodate as the ion-exchange site. The authors concluded that the electrode exhibited a near-Nernstian response within the range of 10^−2^–10^−5^ M for PIT with a slope of 55 mV/decade, a fast response time of (<1 min), and stability in the pH range of 3–7. Selectivity tests demonstrated that the electrode was unaffected by most common anions. It was used to directly determine PIT in the range of 5.24 × 10^−5^ to 1.31 × 10^−2^ M, achieving a recovery rate of 98.9% with a standard deviation of 1.4%. α-diols, reduced carbohydrates, and hydrazine were also determined using the same sensor at concentrations of 0.05 mg/mL –2.5 mg/mL by reacting with PIT, achieving a recovery rate of 98.2% with a standard deviation of 1.7%.

The potentiometric sensors proposed for PIT-determination is based mainly on the ion-exchange mechanism with few exceptions, which were based on ionophores; all these sensors exhibited near-Nernstian slopes within the concentration range of 2–4 orders of magnitude, with detection limits down to micro-molar concentration. Despite the difficulty in potentiometric determination of PIT because of the previously mentioned reasons, these sensors were successfully applied for PIT determination with high accuracy and precision. In addition, these sensors were applied successfully for indirect determination of other organic analyte species, depending on the chemistry and kinetics of PIT, which facilitates the determination of these organic species in food, industrial products, and others. Almost all previously reported potentiometric methods for PIT determination were based on ion-exchange mechanisms, which restrict the selectivity of these methods. In addition, oxidation reactions within the measurement cell caused sensor fouling and irreversibility of responses.

Other potentiometric methods using perchlorate-selective electrodes and including PIT were reported for the determination of other analytes (Table 5). A potentiometric determination of PIT with a perchlorate-selective electrode was conducted based on the reaction of PIT with amino alcohols, where the rate of reaction was monitored (Efstathiou and Hadjiioannou, 2002a). The Mn(II)-catalyzed reaction of PIT and triethanolamine in the presence of trinitriliotriacetic acid (NTA) was found to be rapid, and this method could be used to determine Mn by measuring the time required for a 10 mV potential change with the application of the potentiometric titration method using a perchlorate-selective electrode. Mn could be determined within the range of 0.2 g–2 g with a relative error of approximately 1% within 15 s–100 s; it was successfully used in the analysis of non-ferrous alloy samples and presented good accuracy (Efstathiou and Hadjiioannou, 2002b).

**TABLE 5:** Potentiometric and PIT-based analytical methods for the determination of metal ions, anions, and organic compounds, highlighting the electrode systems, concentration ranges, response characteristics, and analytical applications.

Analyte	Electrode/Method	Concentration range	Response time	Application	Ref.
Mn(II)	PIT/triethanolamine/nitrilotriacetic acid	0.2 µg–2 µg	15 s–100 s	Mn-determination in non-ferrous alloys	Efstathiou and Hadjiioannou (2002a)
Mn(II)	Pyridoxine-acetylacetone/PIT	40 ng–240 ng	-	-	Efstathiou and Hadjiioannou (1977)
Glucose	ClO_4_^−^-ISE	7 mg–54 mg	20 s–140 s	-	Efstathiou and Hadjiioannou (1977)
EDTA/metals	PIT/diethylaniline	7.5 × 10^-6^ M–1 × 10^-3^ M (EDTA); 10^−6^ M–7 × 10^-4^ M (metals)	-	Catalytic titration of Zn, Cu, Hg, and Pb	Hadjiioannou et al. (1977)
ClO_4_^−^/PIT/others	Crystal Violet/Brilliant Green/PVC	10^−6^ M–10^−1^ M (ClO_4_^−^); 10^−6^ M–10^−2^ M (PIT)	<1 min	Detection of Glu, Ser, Man, Phe, and Thr	Negussie et al. (1995)
Glycols (ethylene, propylene, and butylene)	ClO_4_^−^-ISE/PIT oxidation	1.4 × 10^-3^ M–7.0 × 10^−3^ M	15 s–150 s	-	Efstathiou and Hadjiioannou (2002b)
Ethylene glycol	ClO_4_^−^-ISE (direct)	10^−1^ M–10^−5^ M	-	-	Efstathiou et al. (2002)

Another method was developed for microdetermination of Mn(II) based on the catalysis by manganese on pyridoxine–acetylacetone reaction in the presence of PIT (Efstathiou and Hadjiioannou, 1977). The analytical procedure consisted of the automatic measurement of the time required for a 10-mV potential variation using the perchlorate-sensitive electrode. Mn was successfully detected in range of 40 ng–240 ng with relative error of approximately 2%–4%. Thus, these two studies confirmed the involvement of PIT-mediated catalytic reaction in trace-metal determination. These two applications for Mn determination using perchlorate-selective electrode applying PIT were very interesting in terms of their simplicity, accuracy, and sensitivity. The interaction of PIT and carbohydrates was also analyzed using perchlorate-selective electrode (Efstathiou and Hadjiioannou, 1977). A new parameter (PIT index) was developed, and this value was found to be dependent on the molecular configuration and stereo-arrangement of carbohydrates, which made it useful in the detection of small quantities of pure compounds. The kinetic automatic method for the determination of glucose was subsequently developed in a linear range of 7 mg–54 mg. The analytical potentiometric response was approximately 20 s–140 s and yielded good accuracy of approximately 0.4% and 0.8% relative error (Efstathiou and Hadjiioannou, 1977). However, the upper limit of 140 s is relatively slow and may limit suitability for rapid or real-time analysis compared with faster electrochemical methods.

Later, a perchlorate-selective electrode was successfully used as an endpoint sensor in automated semi-automated catalytic titration of EDTA and several metal ions (Zn, Cu, Hg, and Pb) based on the reaction of PIT with diethylaniline as the indicator (Hadjiioannou et al., 1977). The method achieved the detection limit for EDTA within 7.5x10^-6^–10^−3^ M, and for Zn, Cu, Hg, and Pb, the limit was within 10^−6^-7x10^-4^ M. Good repeatability and errors were observed in the range of 1%–2%, which showed that PIT-responsive perchlorate electrodes were useful in complexometric and catalytic analysis (Hadjiioannou et al., 1977).

Furthermore, liquid membrane sensors incorporating Crystal Violet-perchlorate and Brilliant Green-perchlorate ion-association complexes within PVC matrices have been successfully developed (Negussie et al., 1995). The electrodes had good response to perchlorate and PIT within the concentration range of 10^−6^ M–10^−1^ M, response time less than 1 min, and nearly Nernstian slopes. These electrodes have also been successfully used for the indirect determination of glucose, mannitol, serine, ephedrine, and threonine by recovering values of approximately 100% and reasonable precision.

An automated potentiometric kinetic method was then developed for the determination of ethylene glycol, propylene glycol, and butylene glycol by their oxidation using PIT with the monitored residual PIT using a perchlorate-selective electrode (Efstathiou and Hadjiioannou, 2002b). Relative errors were as low as approximately 0.7% for the determination of substances within the concentration range 1.4x10^-3^ M–7.0x10^−3^ M, and the responses were recorded within 15 s–150 s. However, the response time varied considerably (15 s–150 s), with the upper limit being relatively slow for automated or high-throughput analysis. Despite this, the approach demonstrates good applicability for glycol determination with acceptable accuracy and operational simplicity.

### 5.2 Amperometric methods for periodate detection

Amperometry is one of the most useful electrochemical techniques for PIT-detection because of its high sensitivity, quick response, easy operating procedure, and selectivity against other electroactive species in the matrix. This technique depends on the measurement of current produced by the oxidation or reduction of PIT at the working electrode, which is modified with catalytic or conductive materials. Under suitable potentials, the measured current is proportional to the concentration of PIT. Compared with potentiometric methods, amperometric sensors are more sensitive and more readily developed with lower detection limits and faster response time, especially the amperometric sensors based on nanostructured electrode materials (Salimi et al., 2006a; Salimi et al., 2007). However, amperometric determination of PIT exhibited some problems such as low selectivity in real samples, electrode fouling from byproducts of the reaction, signal interference from oxygen, small lifetime of electrode due to instability of chemical modifiers, and multiple preparatory steps for the working electrode or the measurement solution (Salimi et al., 2006a; Salimi et al., 2007).

Recently, significant improvement has been achieved on the analytical performances of amperometric PIT sensors due to substantial developments in nanotechnology and electrode surface engineering (Table 6). Nanomaterials including carbon nanotubes, graphene and its derivatives, nanoparticles of metal, conductive polymers, and metal oxides were widely applied to enhance electron-transfer kinetics, increase the active surface area, decrease overpotential, and stimulate catalytic activity toward PIT-reduction (Salimi et al., 2006a; Salimi et al., 2007; Salimi et al., 2010). This led to very sensitive PIT detection at micromolar and nanomolar levels with widely linear concentration ranges, very fast response times within seconds, and satisfactory operational stability and reproducibility (Salimi et al., 2007; Salimi et al., 2010). In addition to that, the amperometric method is well-compatible with the FIA system.

**TABLE 6:** Overview of the voltammetric and amperometric sensing methods for periodate (PIT) ion detection using modified electrodes.

Analyte	Electrode and modification	Method	Concentration range	Detection limit (DL)	Ref
PIT	GCE/SWCNT/Ir (ppy)_2_ (phen-dione)	Amperometry	1 μM–20 μM; 20 μM–450 µM	0.6 µM	Shamsipur et al. (2013)
PIT	GCE/MWCNT/thionine	Amperometry	1 µM–10 mM	0.4 µM	Salimi et al. (2006a)
PIT	GCE/MWCNT/catalase	Amperometry	1 µM–6 mM	0.15 µM	Salimi et al. (2007)
PIT	IrO_x_ film	Amperometry	up to 80 µM	36 nM	Salimi et al. (2010)
Ethylene glycol	C/SiW12Hex	Amperometry	0.2 mM–5 mM	-	Prodromidis et al. (2001)
PIT	GCE/PLL-GA-SiMo (in H_2_SO_4_)	Amperometry	5 × 10^-6^ M–1.43 × 10^−4^ M	-	Pan et al. (2010)
PIT	Pt wire (rotating electrode)	Static/flow voltammetry	1 × 10^-5^ M–1 × 10^-4^ M (flow)	-	Gökçel and Nişli (1994)
PIT/IO_3_^−^/SO_3_^2-^	Pt/MWCNT-SO_3_^2-^/PB	Cyclic voltammetry and electrochemical impedance spectroscopy	-	8.06 µM (PIT)	Adekunle et al. (2012)
PIT/IO_3_^−^/BrO_3_^−^	GCE/SWCNT/Os(III)LCl_2_	Cyclic voltammetry and amperometry	-	-	Salimi et al. (2008)
PIT/IO_3_^−^/BrO_3_^−^/NO_2_^−^	GCE/MWCNT/vanadium Schiff base	Cyclic voltammetry	-	-	Salimi et al. (2006b)
PIT/IO_3_^−^	GCE/RuO_2_ nanoparticles	Cyclic voltammetry and amperometry	-	0.2 µM (IO_4_^−^)	Chatraei and Zare (2013)
Carbohydrates	Au/Pt/C	Cyclic voltammetry	Nanoscale	-	Torimura et al. (1997)

**FIGURE 8:**
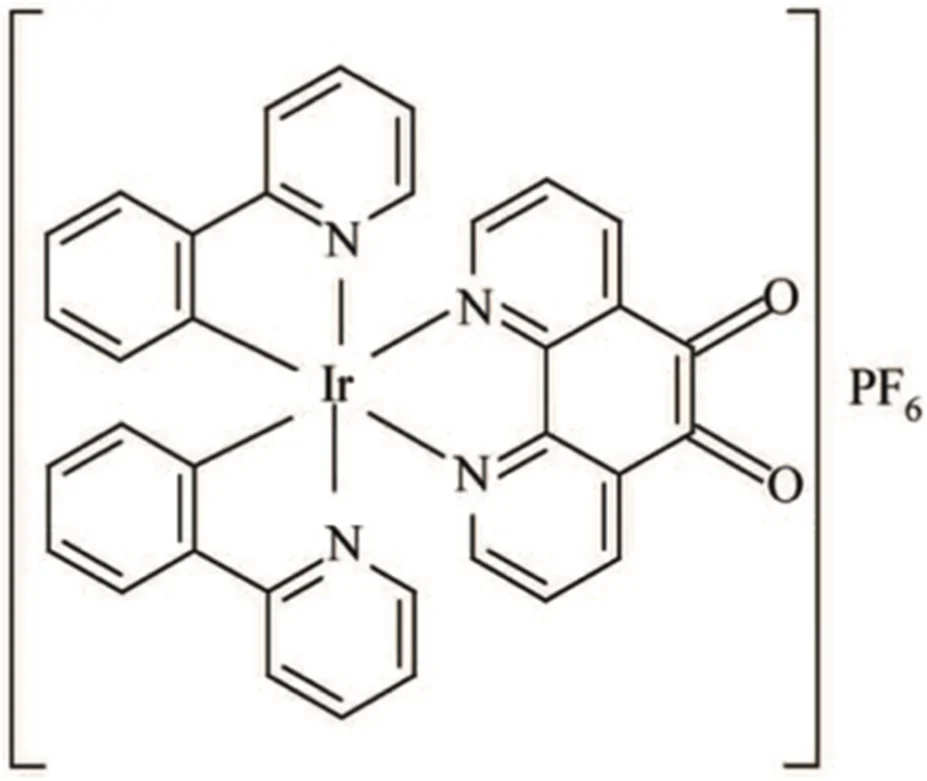
Chemical structure of Ir(ppy)_2_(phen-dione) used for the electrocatalytic reduction of periodate (Shamsipur et al., 2013).

To date, several amperometric methods have been reported, as summarized in Table 6. Shamsipur et al. (2013) immobilized the complex Ir(ppy)_2_(phen-dione) (Figure 8) onto glassy carbon electrode (GCE) via a single-walled carbon nanotube (SWCNT) network to analyze the reduction of PIT. The modified electrode exhibits strong electrocatalytic activity to reduce PIT at 0.200 V and pH 2.0, two linear concentration range between 1 μM–20 μM and 20 μM–450 μM, detection limit of 0.6 µM, and sensitivity of 198 nA M, respectively. This was due to the synergistic effects between SWCNTs and the iridium complex, providing quick electron transfer and catalysis.

Since 2010 to 2012, a series of high-performance amperometric PIT sensors based on multi-walled carbon nanotube (MWCNT) has been reported by Salimi et al. (2006a), Salimi et al. (2007), and Salimi et al. (2010). In the first paper (Salimi et al., 2006a), thionine molecules were deposited between the layers of MWCNT immobilized onto GCE for the electrochemical reduction of PIT. The sensor had a wide linear concentration range from 1 µM to 10 mM with a detection limit of 0.4 µM. In their second paper (Salimi et al., 2007), enzyme catalase was entrapped into the MWCNT matrix to enhance the electrocatalytic activity and efficiency for PIT reduction. The biosensor exhibited linearity in the range of 1 µM–6 mM and a detection limit of 0.15 µM. In the third paper (Salimi et al., 2010), iridium oxide nanoparticles were electrodeposited onto the GCE and coupled with the FIA system to determine PIT. The sensors show good electroanalytical parameters, including sensitivity of 150.6 nA mol, detection limit of 36 nM, and repeatability of 1.5%. This research clearly showed the potential applications of nanostructured electrochemically modified electrodes for PIT detection. However, these methods (Salimi et al., 2006a; Salimi et al., 2007; Salimi et al., 2010) have the disadvantages of the preparatory steps of electrode, limited concentration range, and the use of toxic chemicals. As shown, all methods exhibited very high sensitivity to the sub-micro-molar and nano-molar detection limit, with wide linear concentration range.

In addition, Prodromidis et al. (2001) proposed a modified graphite electrode with a novel Keggin-type silicotungstic acid salt involving hexanediylpyridinium cation (i.e., silicotungstic acid salt with pyridinium-derivative counterion). It was immobilized onto spectroscopical carbon electrodes by physical adsorption from a solution in acetone for achieving chemical modification and then used for amperometric detection of PIT by the reduction to iodide at 0.40 V and pH 4.5. Indirect determination of ethylene glycol in antifreeze solutions was also achieved by using the sensor, where ethylene glycol-mediated PIT reduction was used to develop amperometric signals. A linear relation was found between the cathodic current and ethylene glycol concentration over the range 0.2 mM–5 mM, and excellent recoveries of 98%–101% indicated that it was suitable for analytical application (Prodromidis et al., 2001). The drawbacks of the method include the multiple preparatory steps of the electrode, in addition to the limited concentration range of application.

Interestingly, Pan et al. (2010) designed a modified glassy carbon electrode with glutaraldehyde-linked poly-L-lysine grafted with silicomolybdate (PLL–GA–SiMo) for analyzing the reduction of iodate and PIT in a sulfuric acid medium. The film showed excellent electrocatalytic activity toward the reduction of both iodate and PIT species. Linear working range of 2.5 × 10^−1^ M–1.1 × 10^−3^ M for iodate and 5 × 10^-7^ M–1.43 × 10^−3^ M for PIT were obtained with the remarkable sensitivity of the 18.47 and 1,014.7 AM^-1^, respectively. It was proposed that the sensitivity could originate from the strong redox-mediator capability of silico-molybdate structure and the conductive property of the polymeric film. Amperometric methods can be used for the direct determination of PIT or using it for the indirect determination of industrially relevant organic species. The main disadvantage of the method is the multiple-step preparation of the electroactive species PLL–GA–SiMo.

### 5.3 Voltammetric methods for periodate detection

Voltammetric analysis is a fast, sensitive, and cost-effective method for analyzing PIT in contrast to spectrophotometric and chromatographic approaches (Table 6). This is because the IO/IO redox couple is electroactive, and the potential for electrochemical analysis of the analyte is achievable using cyclic voltammetry (CV), differential pulse voltammetry (DPV), square-wave voltammetry (SWV), stripping voltammetry, and hydrodynamic voltammetric methods. The high sensitivity, speed at which electron transfer can be monitored, and minimal sample requirement and analysis time make voltammetry very suitable for application in environmental, pharmaceutical, and industrial analysis (Abdel-Aziz et al., 2025; Majeed et al., 2022). This method is also useful in studying the mechanistic behavior and reduction pathways of PIT using modified electrodes. Compared to traditional analytical methods, voltammetric analysis has the advantage of lower cost, quicker reaction time, miniaturization, and portability. However, challenges still exist in relation to long-term electrode stability, reproducibility in nano-material synthesis, and surface fouling and matrix interference in real samples. It is expected that portable voltammetric sensors will continue to be developed with advancements in the materials of nano-structured electrodes and with integrated microelectronic systems that can offer real-time measurements in environmental, pharmaceutical, and industrial settings.

To date, highly sensitive and selective detection of PIT through voltammetry is significantly improved using catalytic electrode modifiers, such as transition-metal oxides, Prussian Blue films, conducting polymers, carbon nanotubes, graphene materials, and metal-complex catalysts (Adekunle et al., 2012; Salimi et al., 2006b). By the modification of the electrode surface with these catalytic species, the reduction of PIT can occur at more positive potentials, the electron-transfer rate is increased, background current is decreased, and the stability of the signal is improved. Modified electrodes can detect PIT over a wide linear concentration range down to sub-micro- and nano-mole levels with great stability and efficiency (Adekunle et al., 2012; Chatraei and Zare, 2013; Salimi et al., 2006b). Most methods are carried out in a slightly acidic supporting electrolyte medium such as phosphate buffer, sulfuric acid, or acetate buffers to ensure PIT stability and suitable potential ranges (Adekunle et al., 2012; Chatraei and Zare, 2013; Salimi et al., 2006b). While the use of electrodes can suffer from surface fouling, leading to a need for occasional regeneration and very careful preparation (Adekunle et al., 2012), modern nanostructured and surface-engineered electrodes provide extremely stable and reproducible signals.

Gökçel and Nişli (1994) described the voltammetric determination of static and flow injection analysis of PIT using a platinum-wire electrode. In static voltammetry, concentrations from 4.0x10^-5^ M–4.2x10^−4^ M could be detected at a pH of 7.0–7.4 using a phosphate buffer. In flow injection analysis, varying volumes of 20 µL –100 µL of a PIT solution was injected into a pH 7.0 phosphate buffer stream moving at 1.0 mL/min–1.7 mL/min. Peak currents could be detected at a potential of 0.07 V vs. SCE, and concentration of 1.0x10^-5^ M–1.0x10^−4^ M could be measured with a sample throughput of 15–40 samples/hour (Gökçel and Nişli, 1994). This research is one of the first to successfully utilize flow voltammetry for PIT analysis. The restrictions of this method are its very small concentration range of application and the long retention time of PIT in the flow injection system.

Platinum electrodes modified with Prussian Blue (PB) nanoparticles and sulfonated multi-walled carbon nanotubes (MWCNT) were created to improve the electrochemical determination of iodate, PIT, and sulfite species [106]. These electrodes were characterized using TEM, EDX, CV, and EIS and the Pt–MWCNT–PB electrode was found to have high catalytic efficiency, conductivity, and electron-transfer rate. Limits of detection for iodate, PIT, and sulfite species were 8.30, 8.06, and 4.70 µM, respectively, with a catalytic constant of 1.37x10^6^, 0.34x10^6^, and 5.24x10^7^ cm^3^mol^-1^s^-1^, respectively. This method was also successfully used to quantify iodine in commercial salt samples in the presence of interfering ions (Adekunle et al., 2012). Electrodes suffer from poisoning, fouling, and short lifetime during PIT determination, which were overcome by soaking them in fresh buffer and storing them in a refrigerator for 15 days before reuse.

SWCNTs-modified glassy carbon electrodes with an electrodeposited Os(III)-complex showed stable irreversible immobilization. The modification achieved for 60 s led to a very fast electron-transfer rate, k_s_ = 5.5 and 7.3 s^-1^ at pH 2 and 7, respectively. This electroactive modified electrode was shown to be highly effective for the reduction of bromate, PIT, and iodate species. Detection limits of 36, 38, and 36 nM were achieved for BrO^3-^, PIT, and IO^3−^, respectively (Salimi et al., 2008). Although the sensitivity can be extended to the nano-molar detection limit, the electrode modification includes several steps.

The use of MWCNTs covalently functionalized with a vanadium-Schiff-base complex on a glassy carbon electrode led to a very stable electrode with an electron-transfer constant of 7 s and a transfer coefficient = 0.55. This modified electrode was electrocatalytically active toward the reduction of BrO, PIT, and IO. This method was stable over a large range of pH, making it an excellent candidate for analytical applications (Salimi et al., 2006b).

An electro-sensor developed using rhodium oxide nano-islands electrodeposited on glassy carbon electrode was able to catalyze both iodate and PIT reduction using cyclic voltammetry, chronoamperometry, and amperometry. Electron-transfer characteristics for bare and modified electrodes showed that k_s_ was 9.0 s^-1^, with a transfer coefficient = 0.5. Both iodate and PIT could be detected at limits of detection of 0.9 and 0.2 µM, respectively, demonstrating very high sensitivity (Chatraei and Zare, 2013). The method faces the drawbacks of low reproducibility, in addition to the multi-step sensor preparation process.

Carbohydrates have been detected indirectly via PIT oxidation reactions using voltammetric methods. Carbohydrates could be oxidized by PIT to form iodate, and the iodate was electrochemically reduced. PIT reduction occurred at significantly less positive potential values than iodate reduction using gold, platinum, and carbon electrodes (Torimura et al., 1997). Stable detection using flow injection voltammetry was made using glassy carbon electrodes, which also enabled the formation of coupling with HPLC. It was shown that carbohydrates could be quantified at the nano-mole level using this method (Torimura et al., 1997). Cyclic voltammetric methods for the determination of PIT are advantageous in terms of its selectivity, controlled by the potential and pH, the possibility of different concentration ranges, and very high sensitivity, which may be as low as below 1 nM.

Comparing three different electrochemical methods, namely, potentiometry, amperometry, and voltammetry, each method possesses certain advantages and some drawbacks, and the choice between these methods depends on the application. However, the three methods facilitated the determination of PIT with high accuracy and precision for the different applications. The characteristics, advantages, and disadvantages of these methods are summarized in Table 7, based on the results of Tables 4–6.

**TABLE 7:** Comparison of potentiometry, amperometry, and voltammetry for analytical sensing.

Parameter	Potentiometry	Amperometry	Voltammetry
Sensing element	Ion-exchanger or ionophore-based membrane	Glassy carbon electrode modified with MWCNTs or metal oxide nanoparticles	Glassy carbon electrode modified with MWCNTs or metal oxide nanoparticles
Detection limit	µM range	µM range, with the capability to reach nM levels	Mainly µM range, with potential improvement to nM levels
Sensitivity	Reliable response	Highest sensitivity among the three techniques	Good sensitivity
Simplicity	Very simple and straightforward	Relatively more complex	Simple
Concentration range	1 M to 1 µM	mM to nM	mM to µM
Selectivity	Moderate, with possible interference	High selectivity	Very high selectivity
Response time	Seconds to minutes	Very fast (seconds)	Fast (seconds)
Challenges	Susceptible to interference in real samples, limited reversibility, and relatively long response times	Requires sample preparation and more sophisticated instrumentation	Long-term stability and reproducibility issues

## 6 Analytical applications of periodate as an oxidizing reagent

Periodate (PIT) is widely recognized as a powerful and selective oxidizing agent in analytical chemistry, extending beyond its direct electrochemical determination to a broad range of redox-based analytical applications (Table 8). Its strong oxidative capability, rapid and stoichiometric reaction behavior, and predictable kinetics enable its use in potentiometric titrations, kinetic studies, redox monitoring, and the indirect determination of a variety of organic and pharmaceutical compounds (Pournaghi-Azar and Farhadi, 1997; Zayed et al., 1986). In these systems, PIT functions as both a reactant and an analytical probe, where its consumption or reaction products are monitored to quantify target analytes. These properties make periodate particularly valuable for the determination of phenols, amines, iodides, sulfur-containing compounds, and pharmaceutical substances, especially in complex matrices where direct detection is challenging. The versatility of PIT-based analytical strategies has led to the development of classical titrimetric methods and more advanced potentiometric and kinetic approaches.

**TABLE 8:** Analytical applications of periodate (PIT) as an oxidizing reagent, including the reaction principles, target analytes, and key analytical performance features.

Method	Principle	Target analyte(s)	Medium/Conditions	Analytical features	Ref.
Potentiometric redox monitoring	EMF change during PIT-phenol oxidation	Monohydroxyphenols, nitrobromophenols	Aqueous redox system	Sharp endpoint, high precision, and mechanistic insight (dimer formation)	Zayed et al. (1986)
Potentiometric oxidation titration	TBAPI as oxidant in non-aqueous titration	Phenothiazine derivatives	Chloroform and acidic medium	Micro-scale (5 mg) and pharmaceutical analysis	Pournaghi-Azar and Farhadi (1997)
Indirect iodometric potentiometry	TBAPI–iodide reaction monitoring	Iodide (aqueous and organic)	Non-aqueous/mixed phase	Versatile endpoint detection and adaptable titrations	Pournaghi-Azar and Farhadi (1997)
Kinetic rate method	Reaction rate monitoring of the PIT-iodide system	Iodide	pH 3.51–5.31	Second-order kinetics, k = 5.9 ± 1 L mol^-1^ s^-1^	Marques and Hasty (1980)
Indirect oxidation assay	PIT-mediated oxidation followed by iodometric back-titration	Phenols and nitrophenols	Aqueous	High accuracy and suitable for microdetermination	Zayed et al. (1986)
Indirect spectro-potentiometric hybrid	Redox-induced signal conversion via PIT oxidation	Aromatic amines and phenolic drugs	Aqueous buffered systems	Improved sensitivity and dual-mode detection	Zayed et al. (1986)
Flow/kinetic FIA approach (derived systems)	PIT reaction monitored under flow conditions	Pharmaceutical antioxidants and diols	Flow injection systems	High throughput and fast response	Hartofylax and Efstathiou (1985)
Indirect bioanalytical oxidation	Enzyme substrate oxidation via PIT	Alkaline phosphatase substrates (e.g., p-glycerophosphate)	Aqueous enzymatic media	Indirect enzyme activity monitoring	Hartofylax and Efstathiou (1985)

In a study by Zayed et al. (1986), redox reaction between PIT or iodides and mono hydroxyphenols were monitored using potentiometric techniques. Reaction mechanisms and stoichiometric studies are performed using a standard oxidant solution. This method has been successfully applied for microdetermination of nitrobromophenols by the reduction of unreacted PIT by excess iodide, followed by titration of the remaining excess iodide using standard Hg(II) solution in the presence of a silver amalgam/saturated calomel electrode system. Excellent precision and accuracy were achieved together with a well-defined endpoint in this determination. In addition to that, the authors assumed that the product formed at high oxidant concentration is dimeric.

Pournaghi-Azar and Farhadi (1997) presented a method for potentiometric oxidation titration of phenothiazine derivatives by tetrabutylammonium periodate (TBAPI) in a chloroform medium at a strongly acidic condition. They tried various conditions to make the titration effective and determined the sample weight to be approximately 5 mg using titration with standardized TBAPI solution. The method was effective in determining phenothiazine derivatives even in drugs and medication. Pournaghi-Azar and Farhadi (1997) also proposed a method for the determination of iodides in organic and aqueous phase using TBPI in non-aqueous medium by potentiometric titration. In addition, the reaction between TBAPI and TBAI can be used as the endpoint detection system for acid–base titration in non-aqueous media. Marques and Hasty (1980) performed a kinetic study on the reaction between PIT and iodide, and it was shown that the rate of reaction could be represented by Equation 19:

$$-\frac{d\left[IO_{4}^{-}\right]}{dt}=\left(5.9\pm 1\right)\;L\text{ mo}l^{-1}\;s^{-1}\left[I^{-}\right]\left[IO_{4}^{-}\right].$$ (19)

The reaction proceeded with the determined kinetics at pH 3.51–5.31.

## 7 Exploiting the catalytic effects in analysis

Catalyzed PIT oxidation reactions have been established as a very efficient method for the determination of trace-metals because of their fast reaction rate, simple procedure, good sensitivity, and a relatively low cost of operation. Spectrophotometry, potentiometry, flow injection analysis (FIA), smartphone-based detection, and other new sensors have been successfully developed for trace concentration determination of various transition and noble metals. Based on the strong oxidizing capability of PIT and the easy activation by transition metal ions, many catalytic analysis methods of trace metals have been proposed and used (Table 9). The oxidation rate of PIT is enhanced significantly by trace amounts of Mn, Cr, Fe, Rh, Ir, and V, among others (Efstathiou and Hadjiioannou, 1995; Sklarz, 1967). The catalytic effect caused by these trace metal ions on the oxidation rate of PIT can form the basis of a highly sensitive kinetic analysis method for the determination of the metals at micro-to-nano levels and even at sub-nanogram levels.

**TABLE 9:** PIT-catalyzed analytical methods for trace-metal determination.

Metal ion (catalyst)	Reaction system (PIT-based)	Analytical method	Detection approach	Key advantage	Example/Notes	Ref.
Mn(II)	Oxidation of malachite green or TMB	Spectrophotometry/smartphone sensing	Absorbance- or image-based color analysis	High sensitivity, portable, and low cost	Smartphone-based TMB oxidation for field Mn detection	Shindo et al. (2011) and Apichai et al. (2022)
Fe(III)	Oxidation of methyl violet	Kinetic spectrophotometry	Color decolorization rate	High sensitivity and activator-enhanced response	Uses 1,10-phenanthroline the as activator	Khomutova et al. (2013)
Rh(III)	Oxidation of malachite green	Kinetic spectrophotometry	Reaction rate monitoring	Ultra-trace detection (ng level)	Highly sensitive catalytic oxidation system	Khomutova et al. (2013)
Ir(III) + Rh(III)	Oxidation of sulfarsazene	Flow injection analysis (FIA)	Continuous flow signal response	Simultaneous multi-metal detection	Fast and selective noble-metal detection	Khomutova et al. (2013)
V(V)	Oxidation of methylene blue	Spectrophotometry	Rate enhancement/absorbance change	High selectivity and sensitivity	Detection at µg/mL levels	Khomutova et al. (2013)
Mn oxides (*in situ* formed)	PIT oxidation of organic pollutants	Environmental catalytic degradation	Indirect monitoring of pollutant removal	Enhanced degradation efficiency	Colloidal MnO_x_ acts as the active intermediate	Zong et al. (2023)

Manganese is one of the most widely studied catalysts in PIT systems. Mn(II) ions were found to catalyze malachite green oxidation by PIT, making a sensitive spectrophotometric determination of manganese using portable absorbance detectors possible (Shindo et al., 2011). Catalytic reaction speeds up the dye oxidation, and the rate of change of color was proportional to the concentration of manganese. It can be used for rapid and effective field analysis, with good precision and low detection limits. More recently, an environmentally friendly smartphone-assisted analytical method was developed for the determination of Mn(II) through PIT oxidation of 3,3,5,5-tetramethylbenzidine (TMB) (Apichai et al., 2022). The colored product formed from the catalysis of oxidation was read out using a smartphone camera and digitally analyzed.

Iron (III) ions were also explored to accelerate the oxidation reactions by PIT. One study performed the Fe(III) catalyzing oxidation of methyl violet to determine trace-level iron in an aqueous solution with the help of 1,10-phenanthroline as an activator (Khomutova et al., 2013). It exhibits excellent sensitivity. Other catalytic kinetic methods were also developed based on the acceleration on decolorization of dyes by iron ions. All these methods have found wide application for the trace-metal determination since they offer convenience and are less susceptible to interferences than traditional spectrophotometric method.

Rhodium (III) was reported to catalyze the oxidation of malachite green by PIT, leading to a sensitive kinetic spectrophotometric method to determine Rh at the ng level (Khomutova et al., 2013). Simultaneously, iridium and rhodium were detected by their catalysis on PIT oxidation of sulfarsazene using a flow-injection system, showing its application for the simultaneous determination of noble metal, with high selectivity and fast analysis (Khomutova et al., 2013). Vanadium (V) has also been determined through catalysis on the oxidation of methylene blue by PIT. The reaction rate increased significantly with the presence of V, and determination can be made with high selectivity and sensitivity at g/mL levels (Khomutova et al., 2013).

Other than the application in trace-metal determination, research on the catalytic roles of the metal oxides formed during PIT oxidation reactions was also carried out. It is important to understand the function of these oxides to develop a suitable condition and better catalytic activity to enhance the decomposition of organic pollutants in environmental remediation technology. The colloidal manganese oxide formed during PIT oxidation reactions was analyzed and discussed for its contribution to the degradation of environmental organic contaminants (Zong et al., 2023). It was found that the species play roles as catalysts and active intermediates to enhance the degradation processes.

Compared with traditional analytical methods, PIT-catalyzed systems showed several advantages such as ease of experimental operation, rapid response, economical reagent consumption, and high selectivity and sensitivity. Additionally, their compatibility with portable and miniaturized instruments broadens their application in environmental, pharmaceutical, and industrial quality and biological analysis. Recently, some smartphone-based and portable colorimetric sensors have been reported, which greatly enhanced their application and environment-friendliness, among others. However, some factors such as co-existing catalysts and oxidable substances, strict requirement of pH and ionic strength, and detailed kinetic mechanism of catalysis reactions remain unsolved challenges and would affect the accuracy and precision. The sensitivity is still not high enough for some specific applications, and the matrix effect should be considered.

## 8 Conclusion and future perspectives

PITs constitute a broad and diverse group of strong oxidants playing key roles in analytical chemistry, catalysis, environment science, green synthesis, and industries. With their multifaceted redox chemistry, multiple forms of structure, and pH-dependent nature, the iodic acids possess versatile characteristics that are distinct from other iodine oxy-acids for diverse chemical transformations and electrochemical system operations. Diverse pathways of oxidation involving electron transfer, radical generation, and oxygen atom transfer have enabled the versatile application of PIT-based advanced oxidation processes for the destruction of broad organic pollutants. From the view of environmental science, the PIT-based advanced oxidation processes offer efficient and eco-friendly methods for emerging contaminants, such as pharmaceuticals, dyes, endocrine disruptors, and waste materials from industries, in many conditions under the mild status. The applications of PIT in green chemistry can also promote the cleaner synthesis and synthesis of active pharmaceutical ingredients by virtue of its higher selectivity and lower byproducts compared with conventional oxidants. Electrochemical methods not only expand the application of PIT but also present sensitive electrochemical detection and real-time monitoring of PIT species. With the advanced material of electrodes (i.e., nanostructured and boron-doped diamond electrode), PIT-based electrochemical systems showed high sensitivity and stability compared to conventional oxidants and electrodes. Nevertheless, the large-scale eco-friendly synthesis of PIT, stability and reusability of electrodes, a deep understanding of the mechanism for the formation of reactive species, and the optimization of conditions are still research frontiers that require further efforts. The chemistry of PIT bridges inorganic redox chemistry and application in the environment and industries. Further studies will focus on the electrochemistry approach for green synthesis of PIT and highly efficient electrochemical sensors based on their specific redox chemistry. Although different species of PIT at different conditions are well-known, which contributes to a better understanding of the role of these species in the different chemical processes, there is no complete explanation of the experimental results yet. In addition, the preparation of new ionophores that can be incorporated for the preparation of simple potentiometric sensors for PIT determination should be considered; this will improve the selectivity and reversibility of the sensor to be applied in complex real samples. In addition, there is a lack of comparative study based on the detailed and systematic relation among PIT species, electrochemical performance, catalytic activity, and analytic sensitivity. This knowledge will provide guidance for designing new sensors, catalytic systems, and environmental applications.
